# Supplementary material for: Small AntiMicrobial Peptide with In Vivo Activity Against Sepsis
Source: Molecules. 2019 May 1;24(9):1702. doi: 10.3390/molecules24091702 (PMC6539432; doi:10.3390/molecules24091702)

^{1}H and ^{13}C NMR spectra

^{1}H NMR spectra of methyl 2,4-dicyano-2-(2-cyanoethyl)butanoate 23, 250 MHz, $CDCl_3$

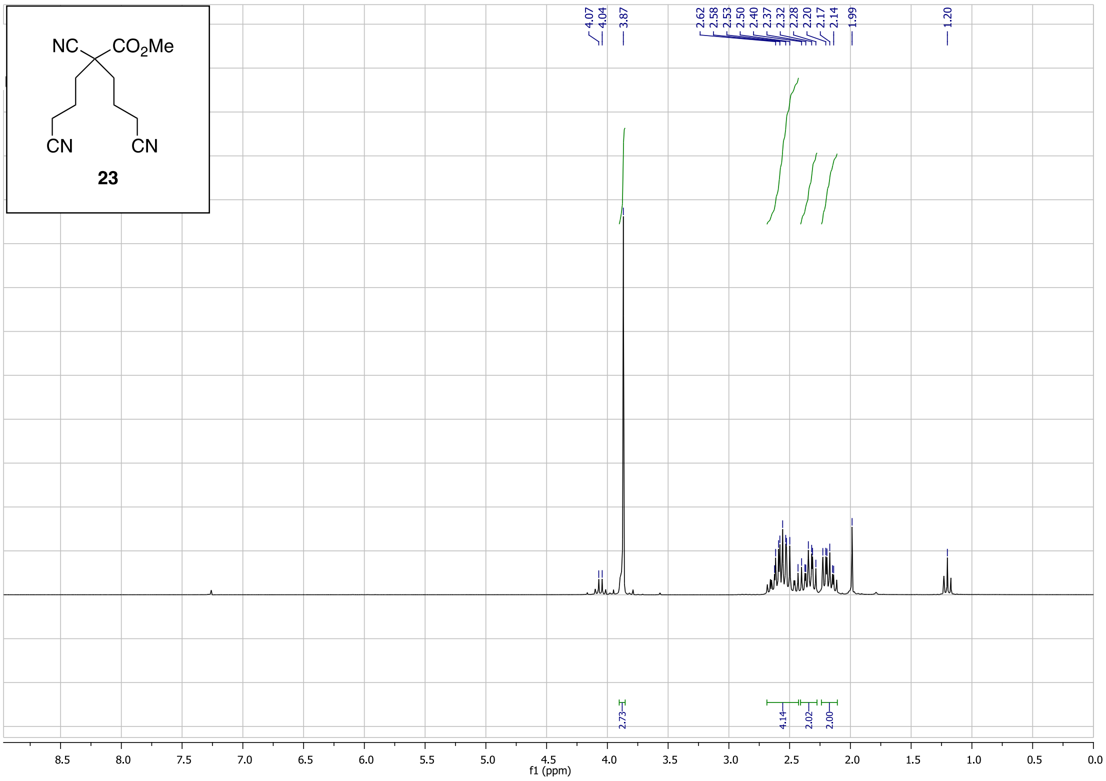

¹³C NMR spectra of methyl 2,4-dicyano-2-(2-cyanoethyl)butanoate 23, 75 MHz, $CDCl_3$

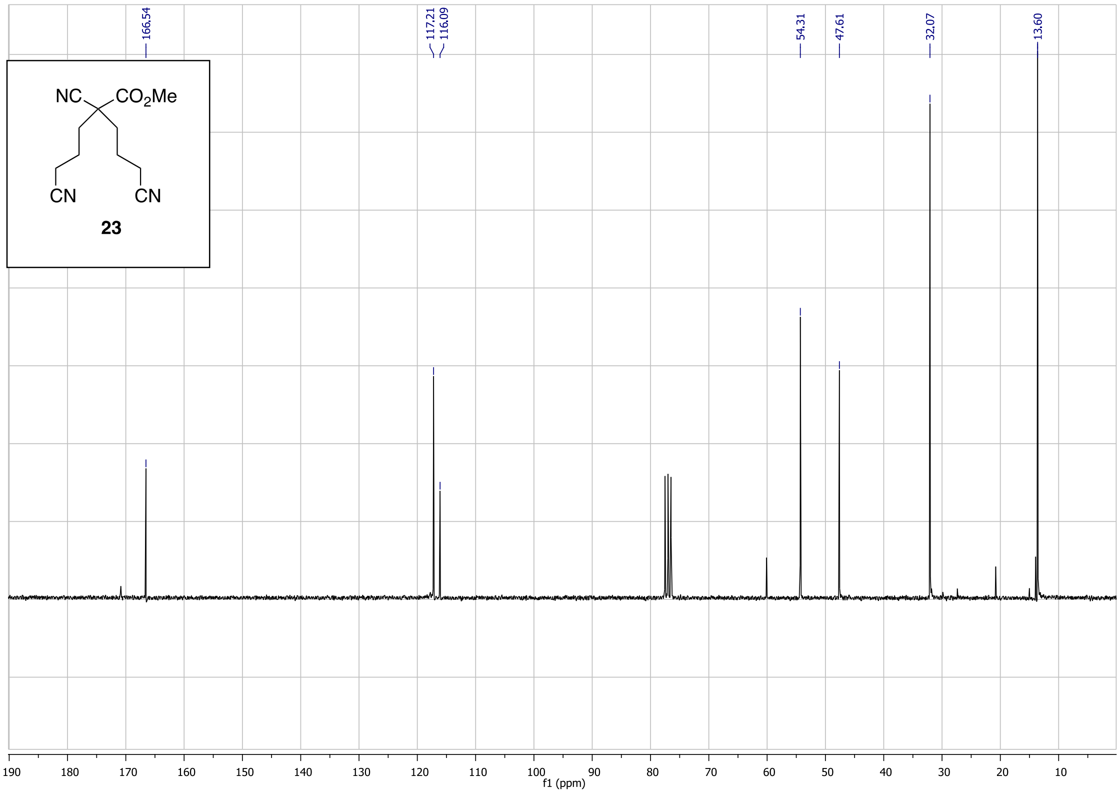

¹H NMR spectra of methyl 2-cyano-4-(Boc)amine-2-(3-(Boc)amine propyl)pentanoate 4, 300 MHz, $CDCl_3$

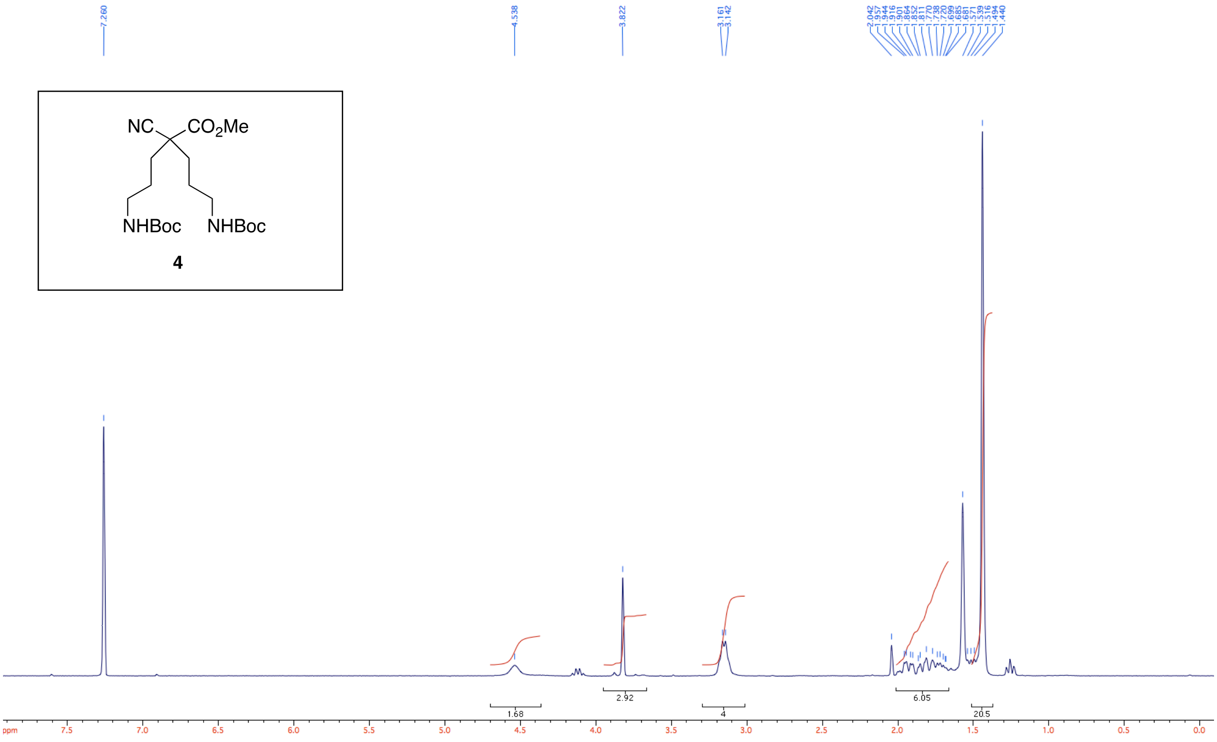

^{13}C NMR spectra of methyl 2-cyano-4-(Boc)amine-2-(3-(Boc)amine propyl)pentanoate 4, 75 MHz, $CDCl_3$

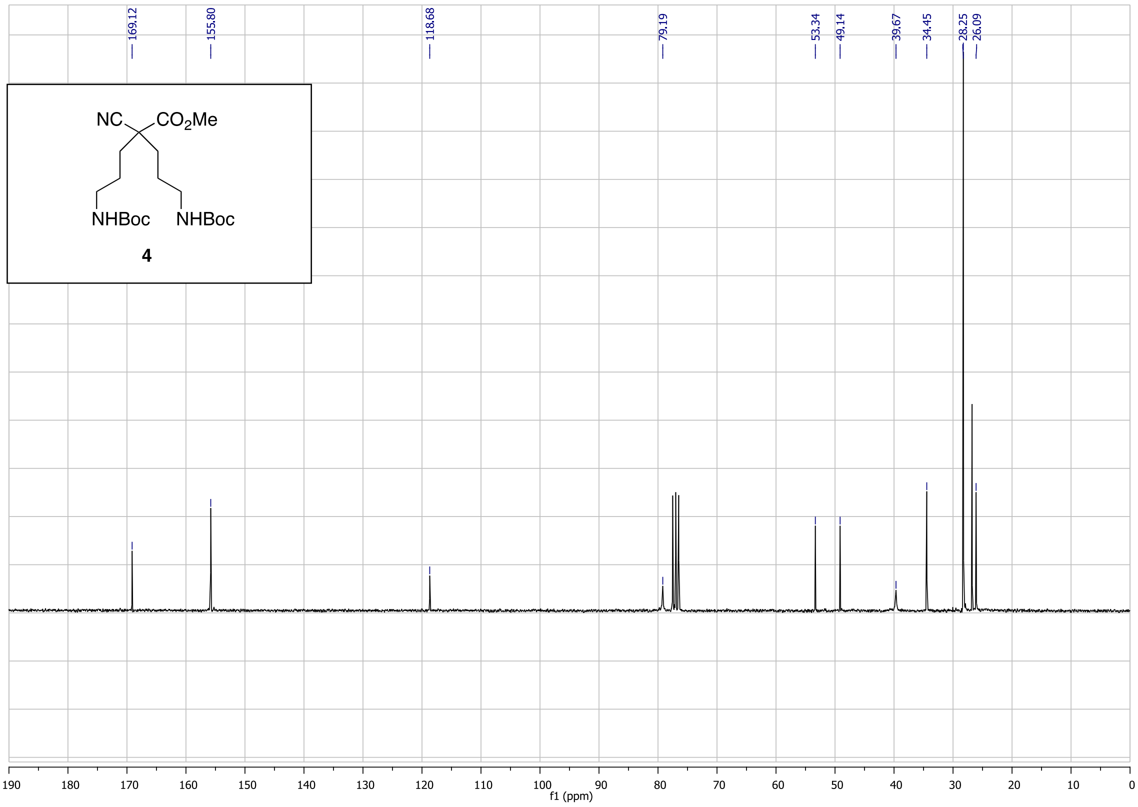

^{1}H NMR spectra of H-$\beta^{2,2}$*h* bis-Orn(Boc)$_2$OMe 1, 300 MHz, CD_3OD

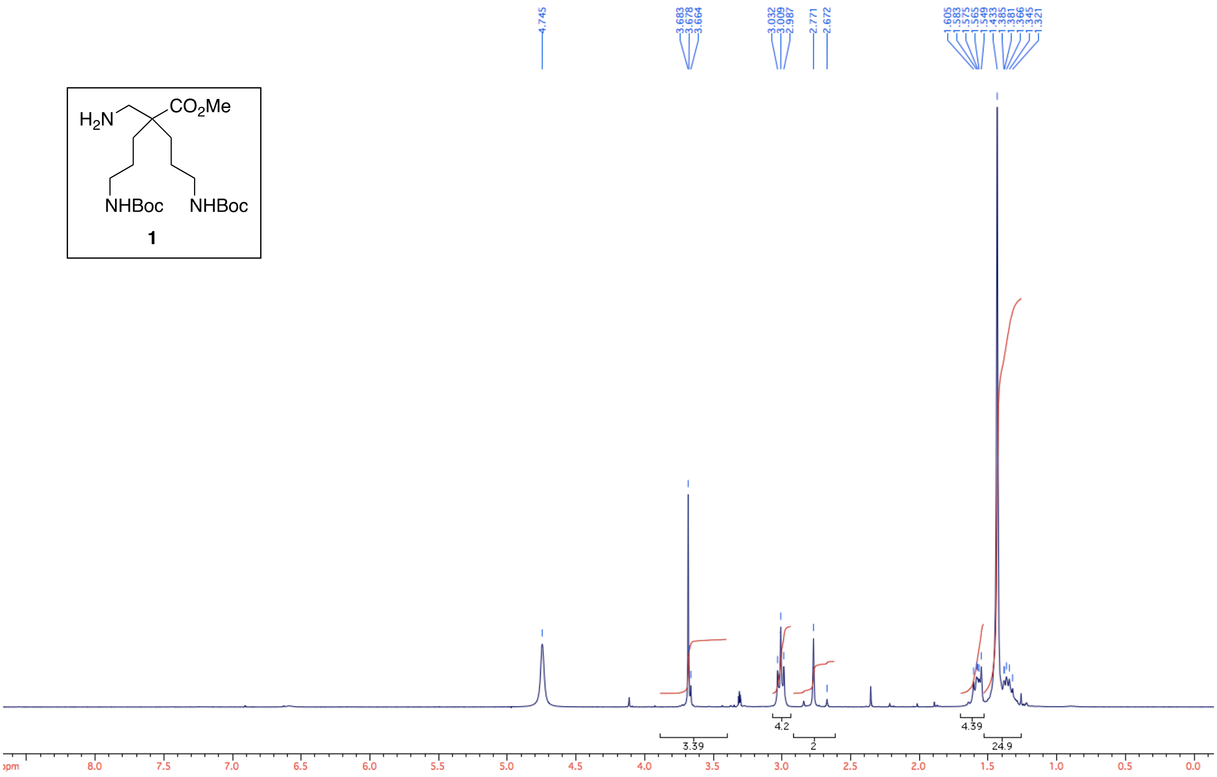

^{13}C NMR spectra of H-$\beta^{2,2}$*h* bis-Orn(Boc)$_2$OMe 1, 75 MHz, CD_3OD

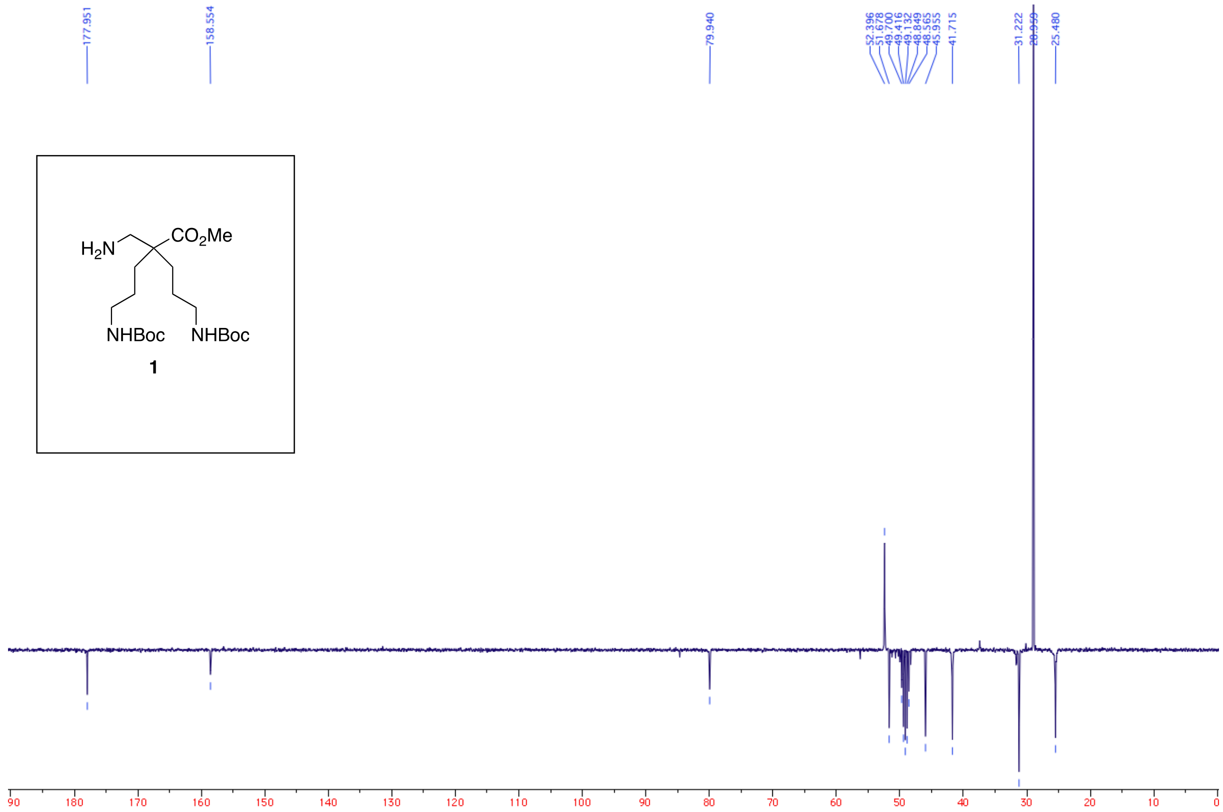

^{1}H NMR spectra of Fmoc β^{2,2} *h* bis-Orn(Boc)$_2$OH 2, 250 MHz, CDCl$_3$

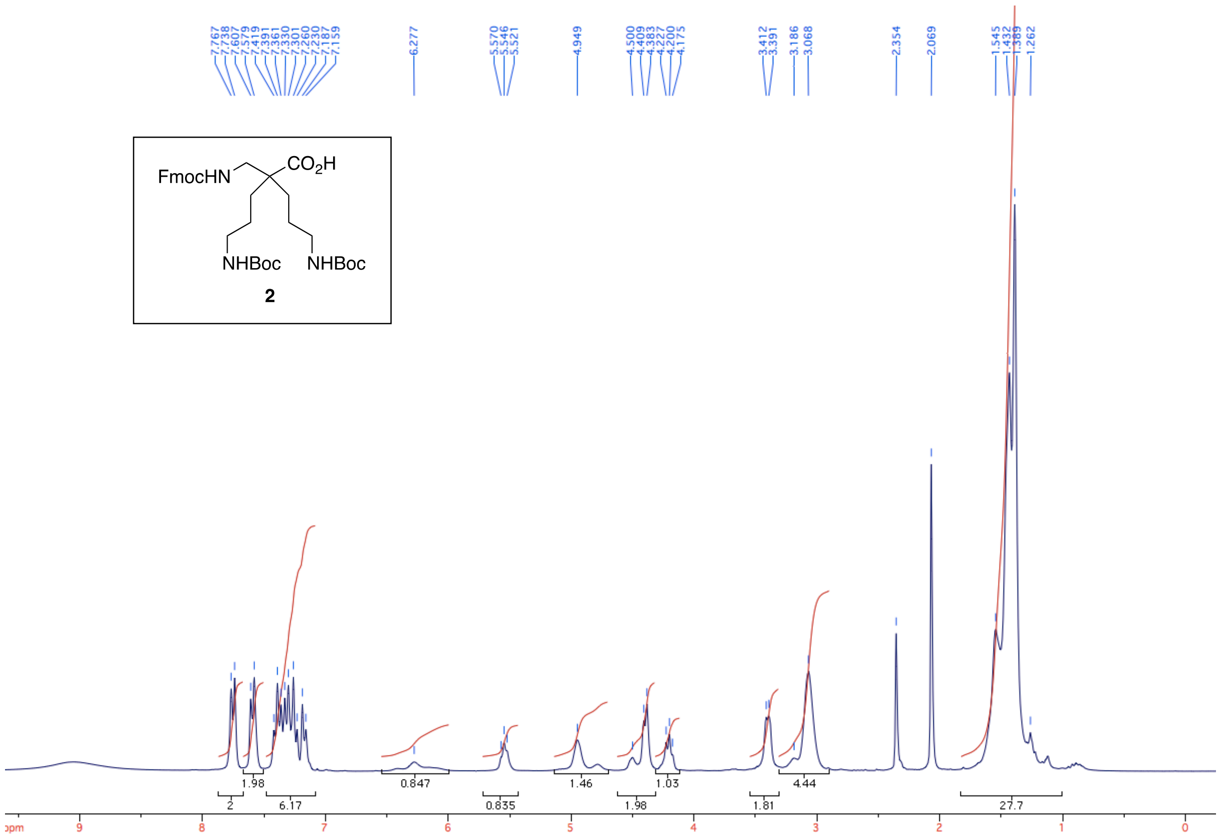

^{13}C NMR spectra of Fmoc β^{2,2} *h* bis-Orn(Boc)$_2$OH 2, 75 MHz, $CDCl_3$

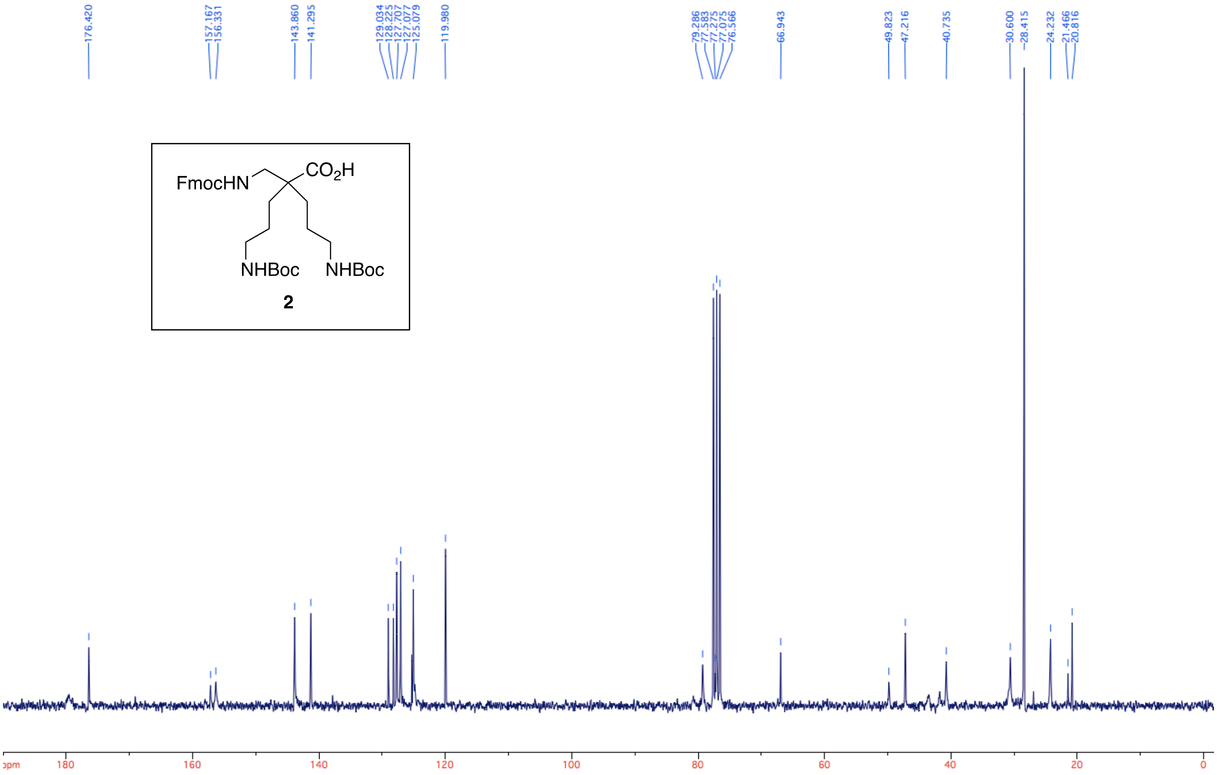

^{1}H NMR spectra of 1-benzyl 3-*tert*-butyl 2,2-bis(2-cyanoethyl)malonate 24, 250 MHz, $CDCl_3$

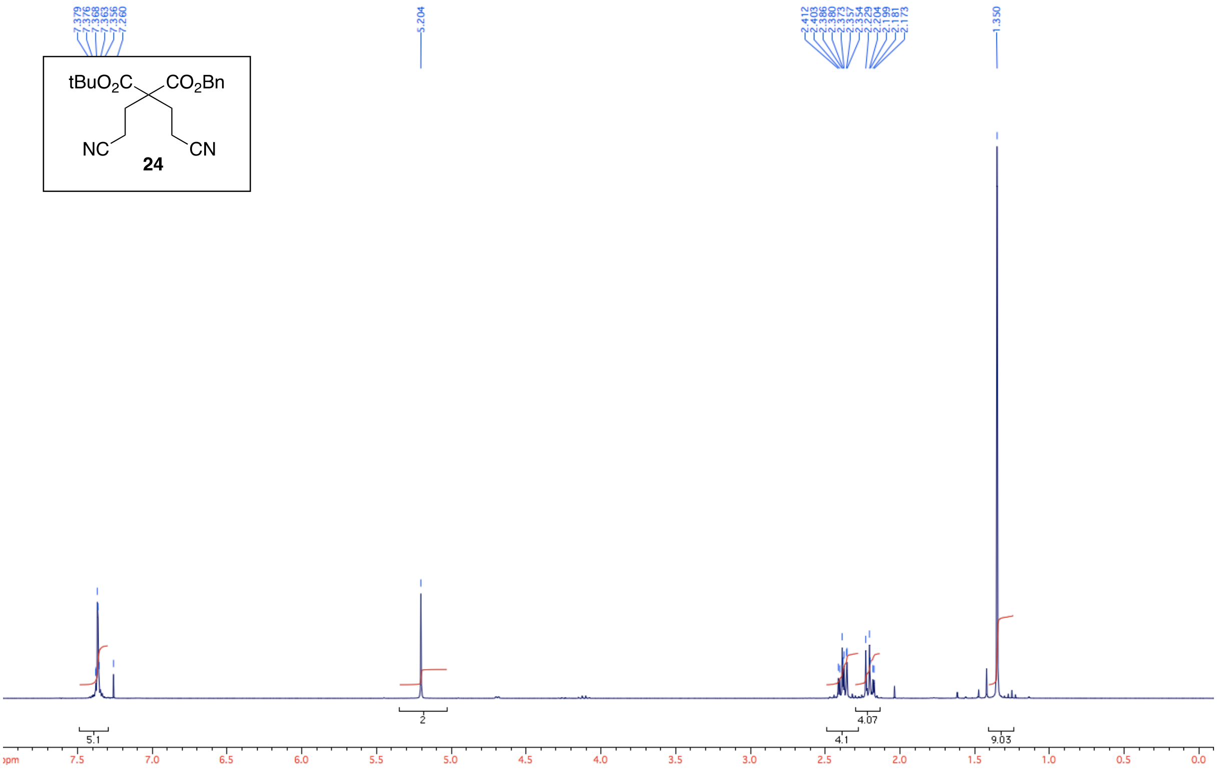

^{13}C NMR spectra of 1-benzyl 3-*tert*-butyl 2,2-bis(2-cyanoethyl)malonate 24, 62.5 MHz, $CDCl_3$

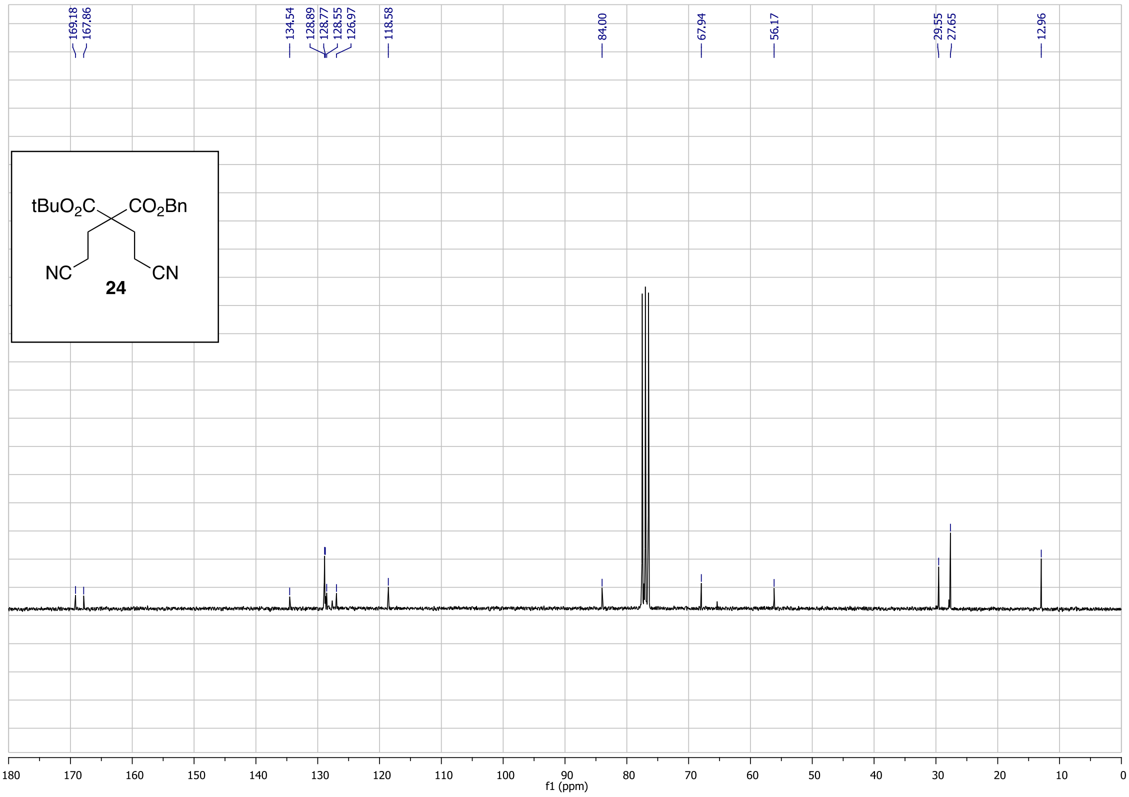

¹H NMR spectra of 2-(tert-butoxycarbonyl)-4-cyano-2-(2-cyanoethyl)butanoic acid 5, 300 MHz, CDCl₃

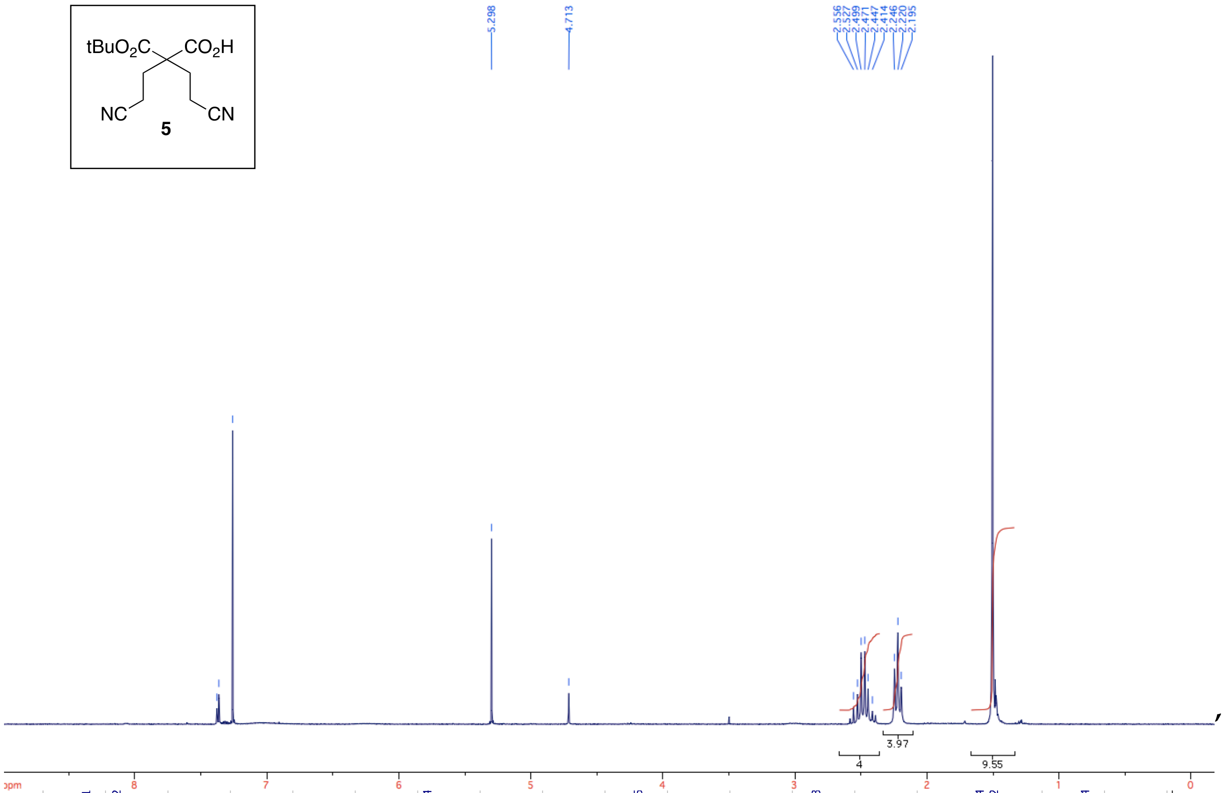

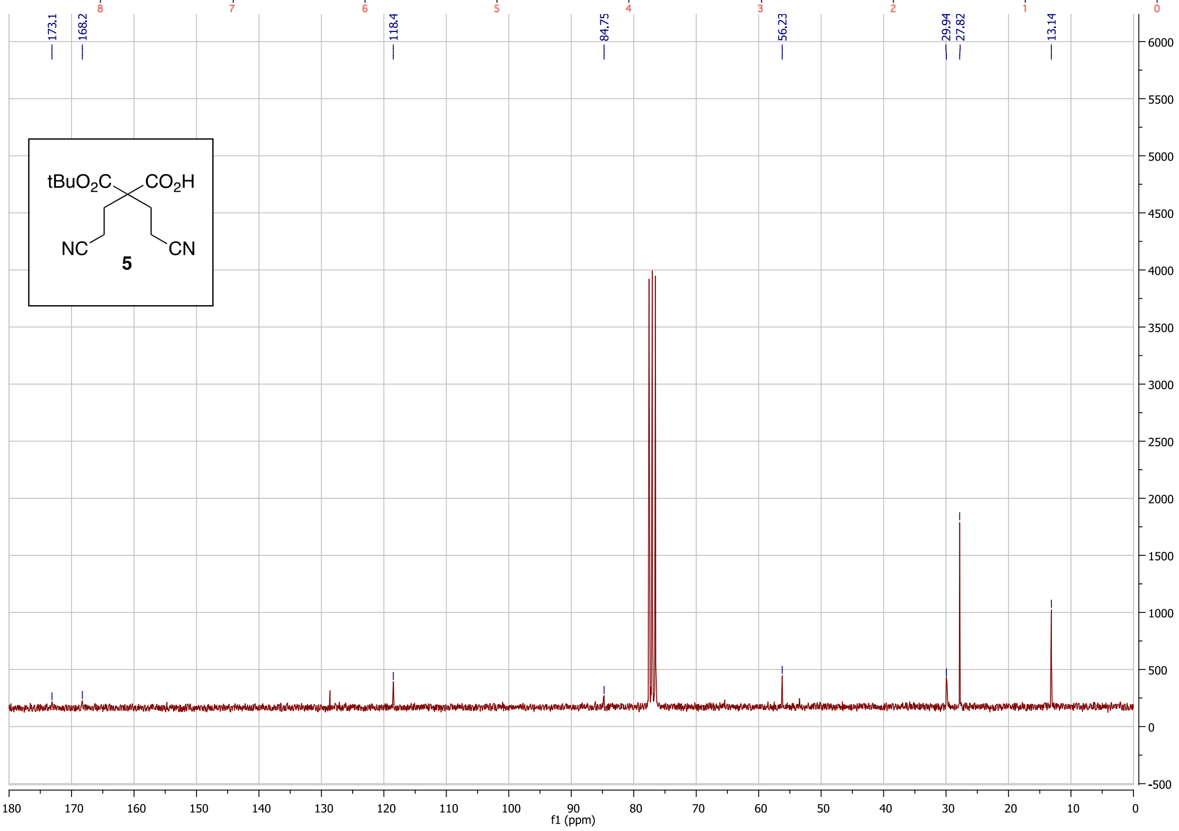

^{1}H NMR spectra of 1-tert-butyl 4-methyl 2,2-bis(2-cyanoethyl)succinate 6, 300 MHz, $CDCl_3$

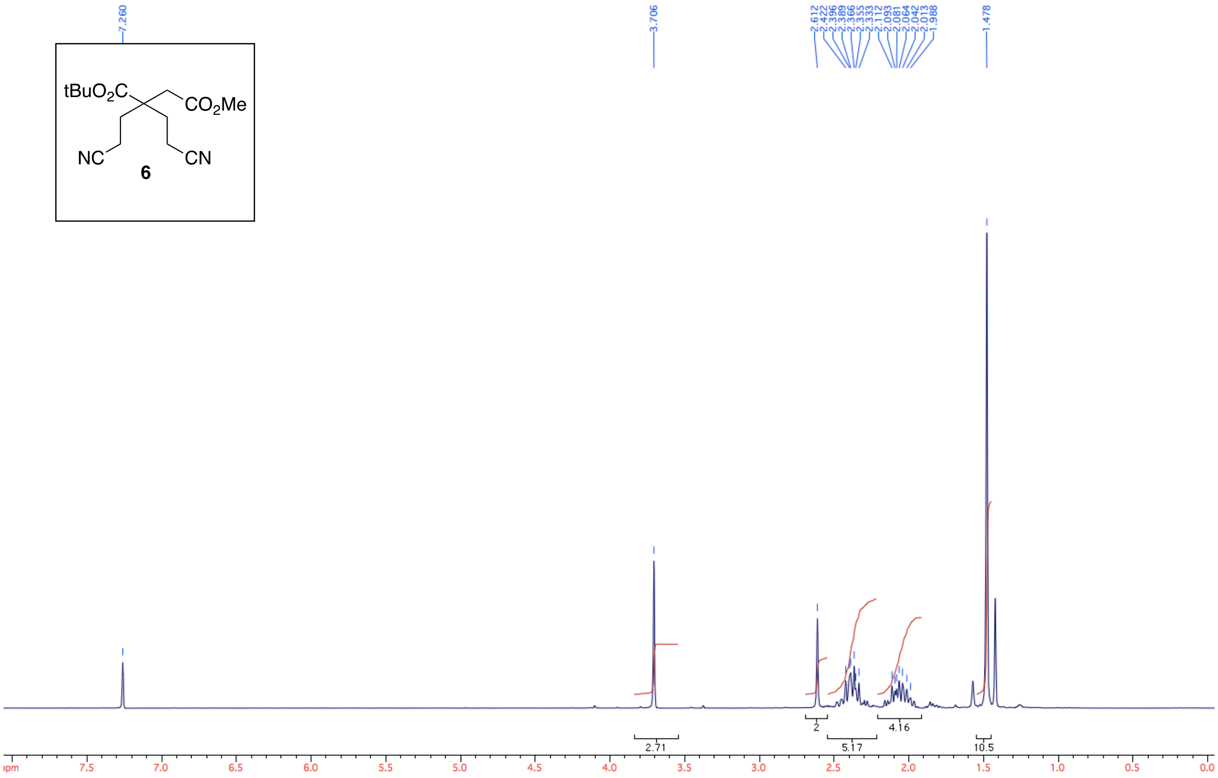

^{13}C NMR spectra of 1-tert-butyl 4-methyl 2,2-bis(2-cyanoethyl)succinate 6, 75 MHz, $CDCl_3$

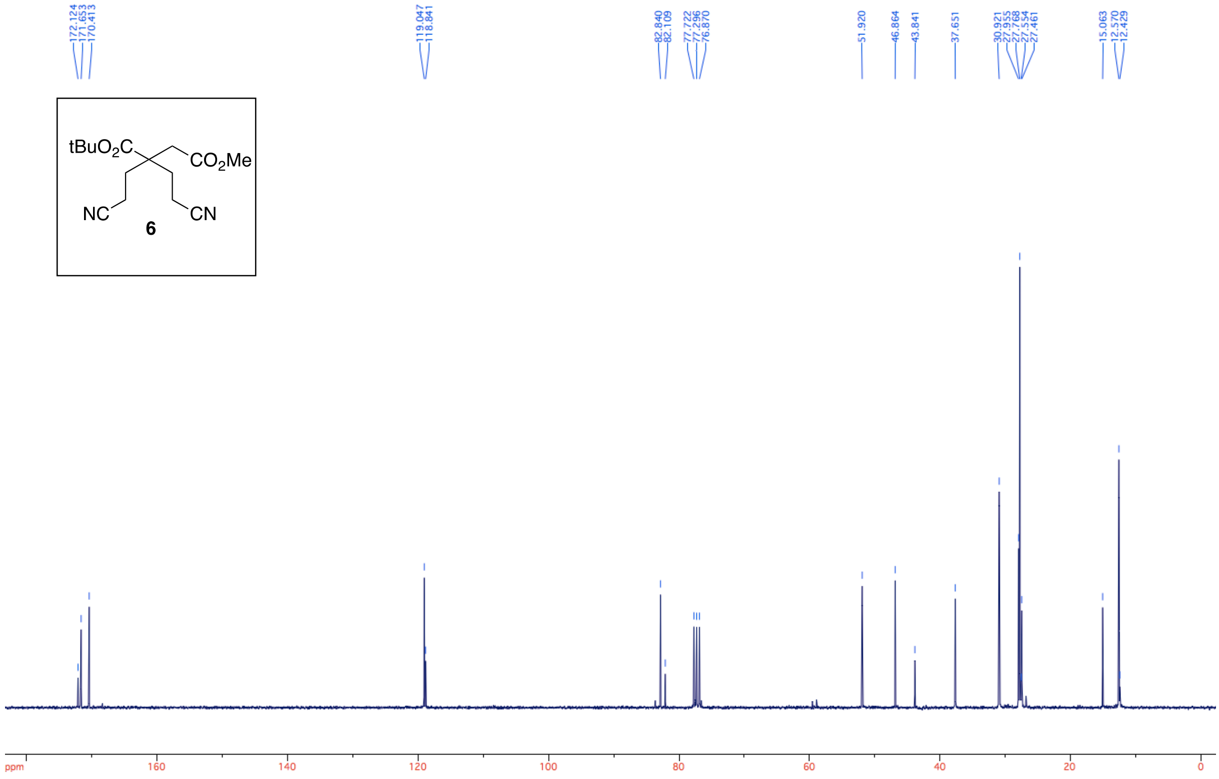

^{1}H NMR spectra of 2,2-bis(2-cyanoethyl)-4-methoxy-4-oxobutanoic acid 25, 300 MHz, $CDCl_3$

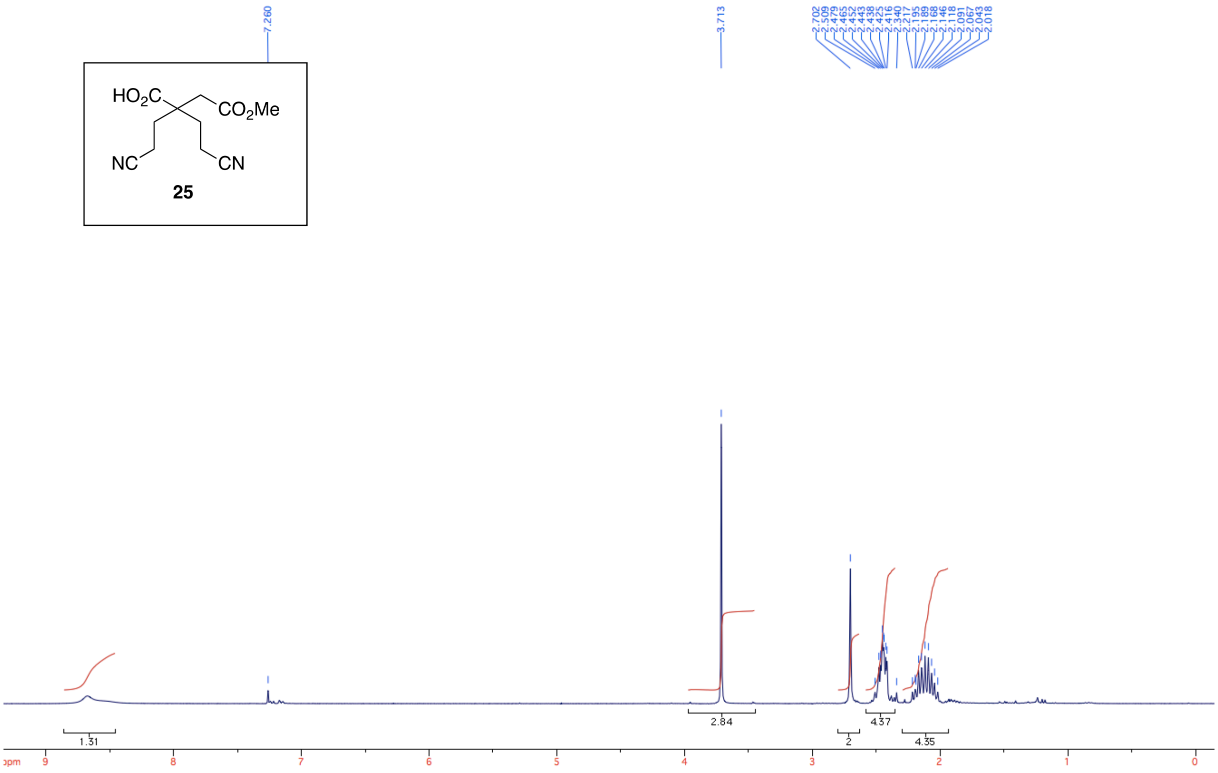

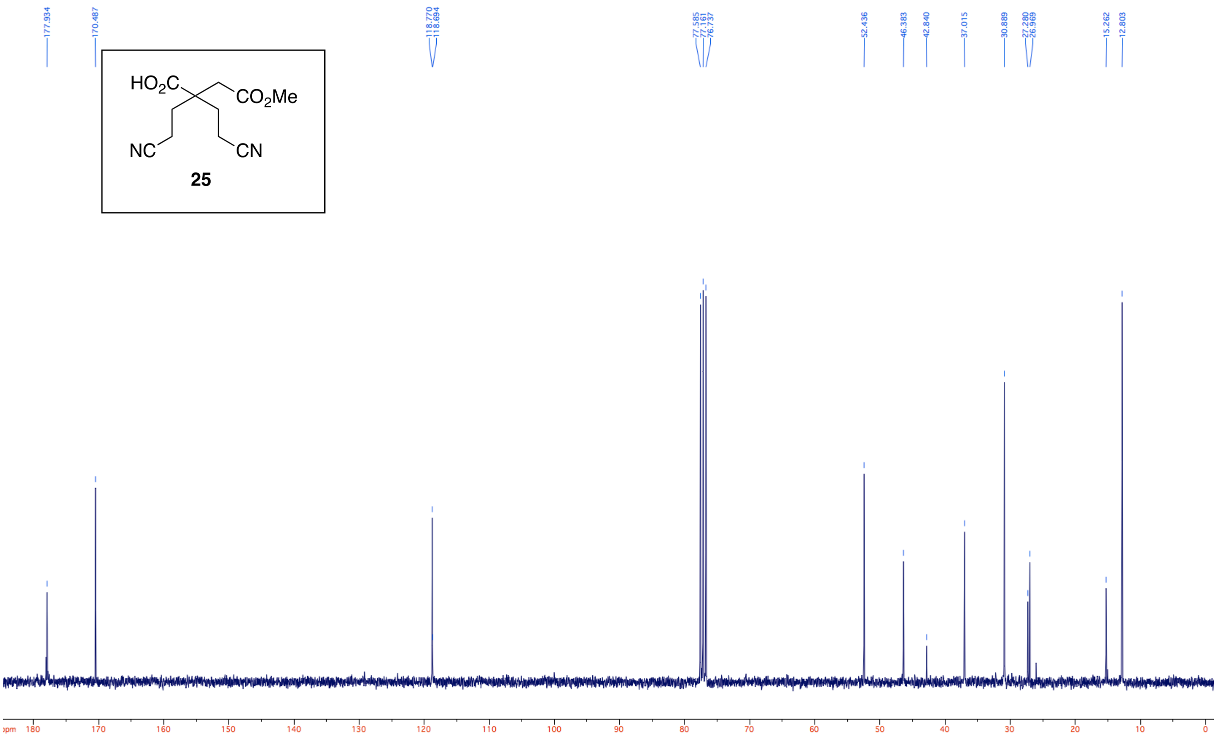

^{1}H NMR spectra of methyl 3-((tert-butoxycarbonyl)amino)-5-cyano-3-(2-cyanoethyl)pentanoate 7, 300 MHz, $CDCl_3$

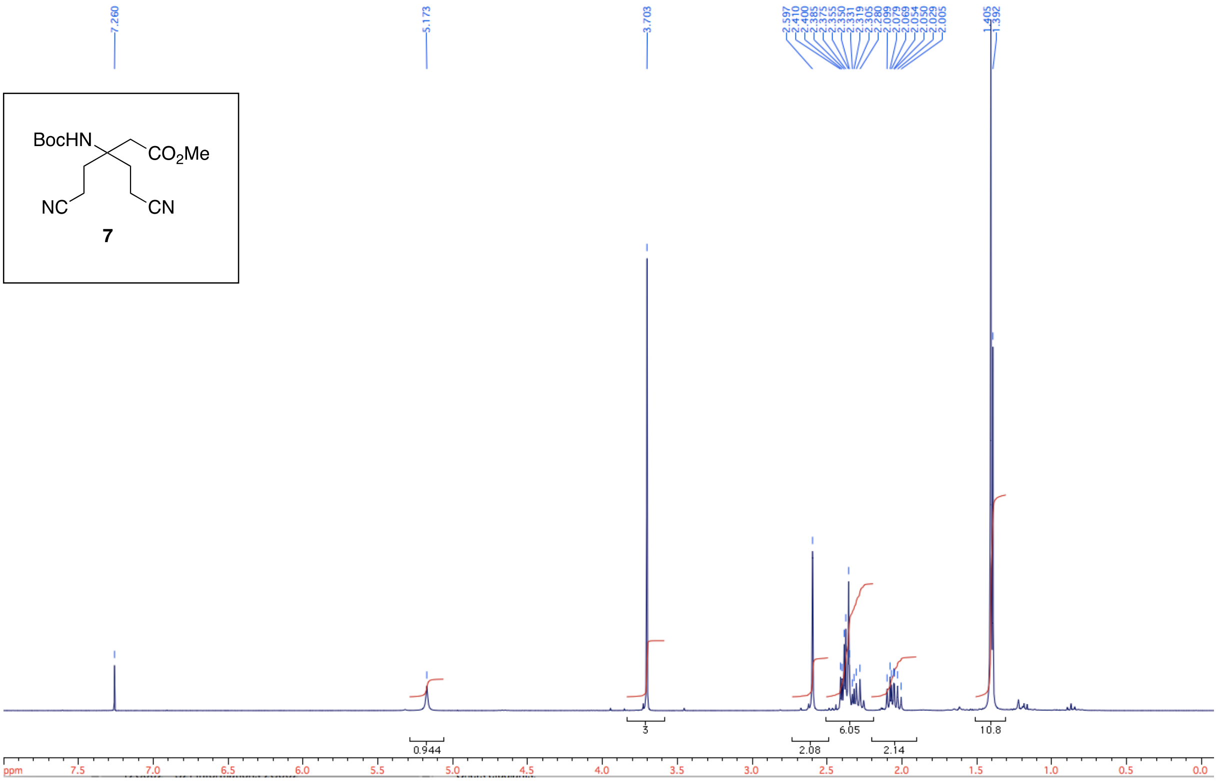

^{13}C NMR spectra of methyl 3-((tert-butoxycarbonyl)amino)-5-cyano-3-(2-cyanoethyl)pentanoate 7, 75 MHz, $CDCl_3$

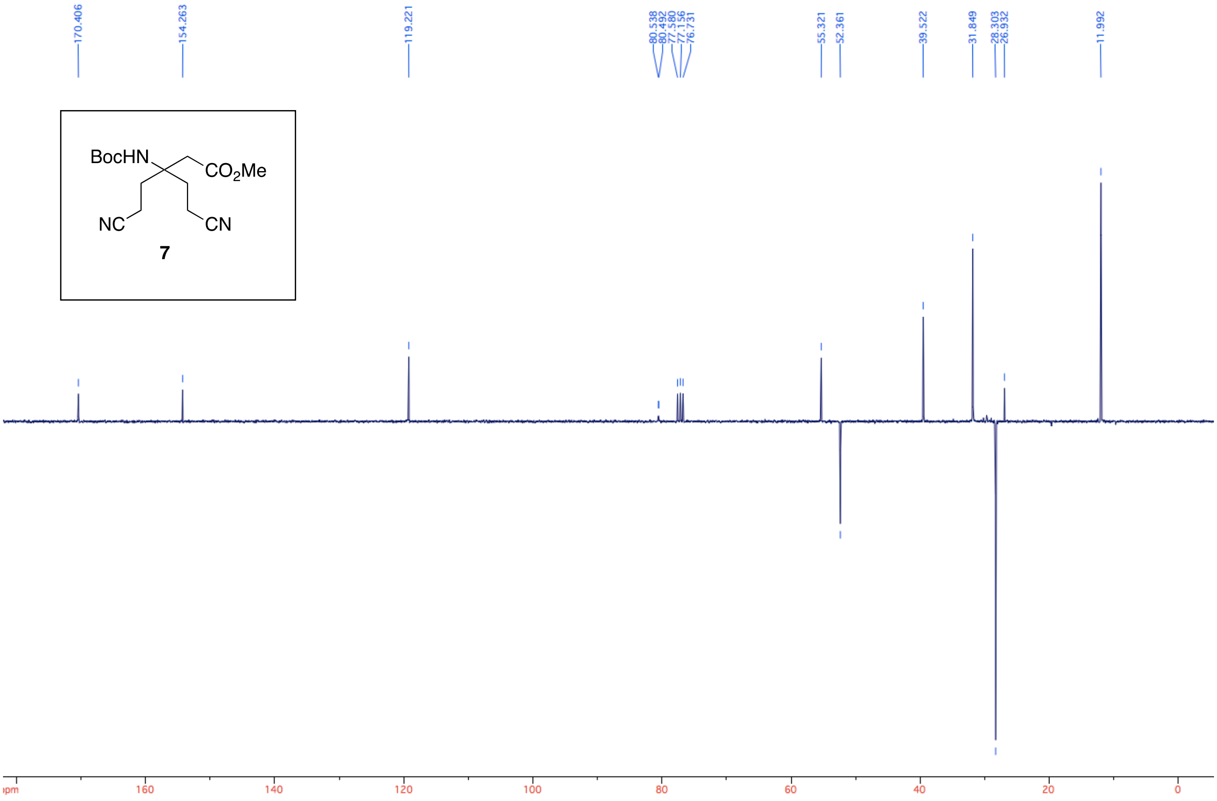

^{1}H NMR spectra of Boc-$\beta^{3,3}$ *h* bis-Orn(Boc)$_2$OH 3, 300 MHz, CD_3OD

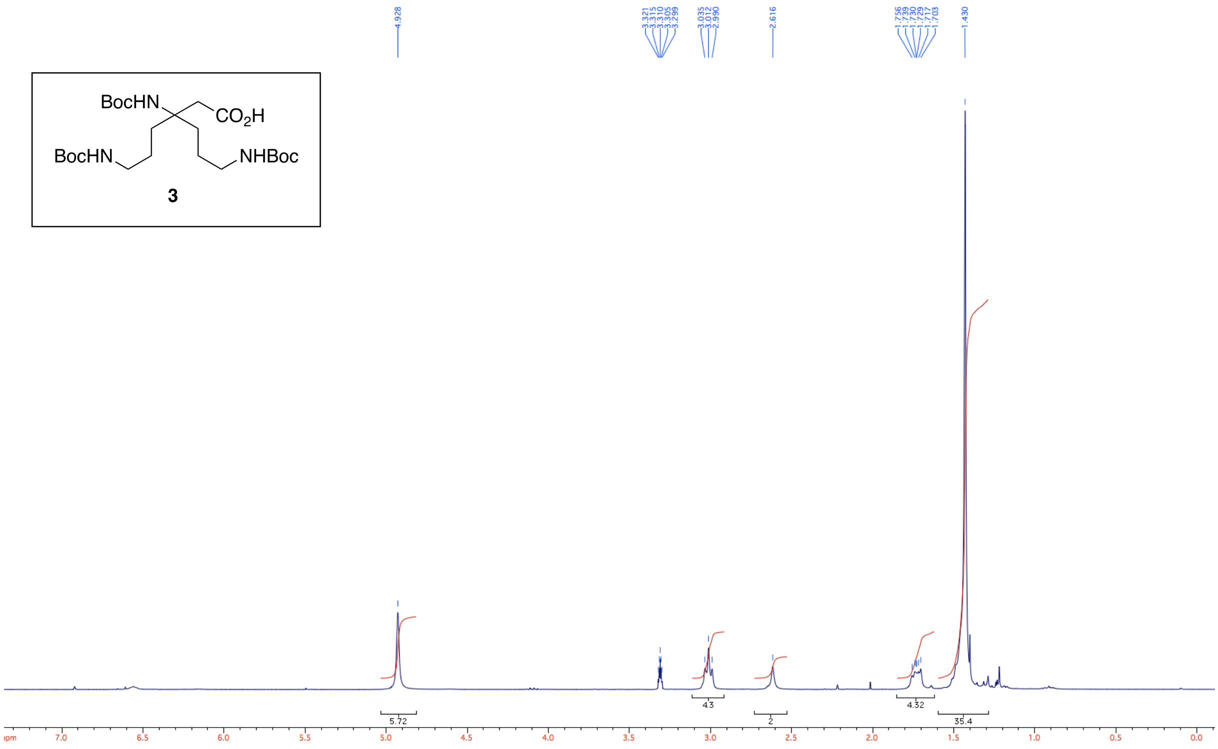

^{13}C NMR spectra of Boc-$\beta^{3,3}$ *h* bis-Orn(Boc)$_2$OH 3, 75 MHz, CD_3OD

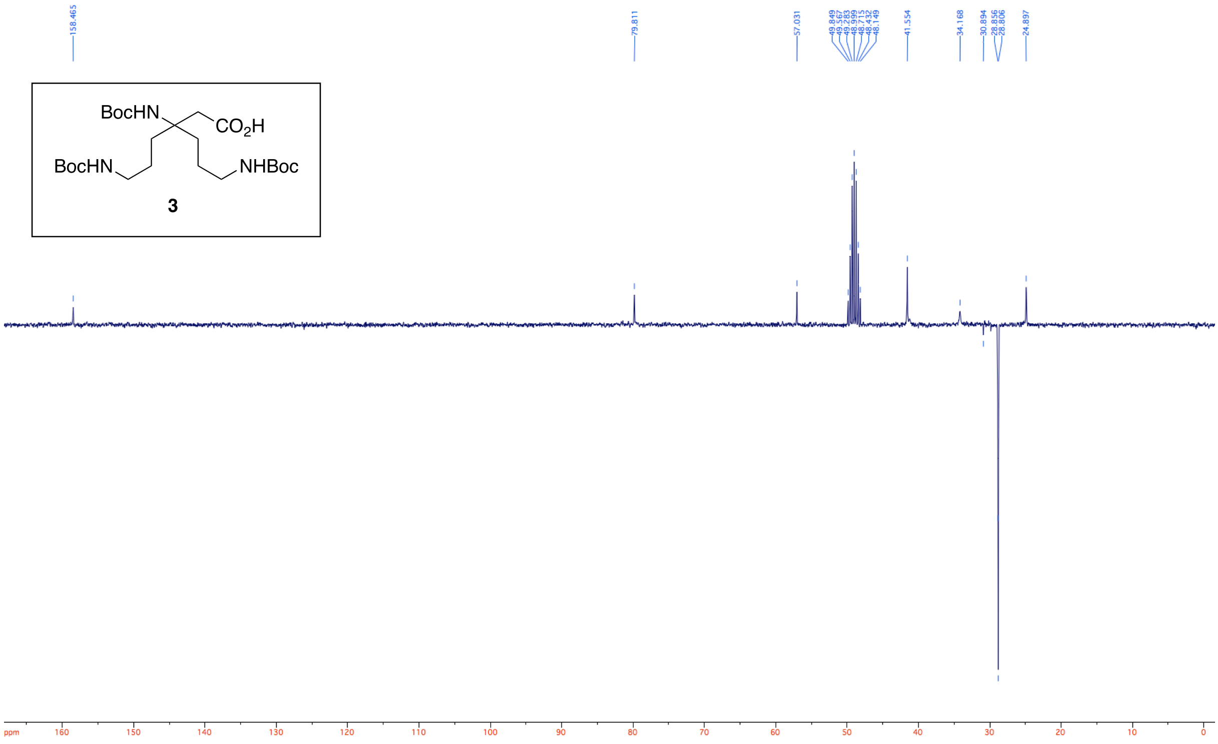

^{1}H NMR spectra of Boc Trp-β^{2,2}*h* bis-Orn(Boc)$_2$-OMe 15, 300MHz, CD_3OD

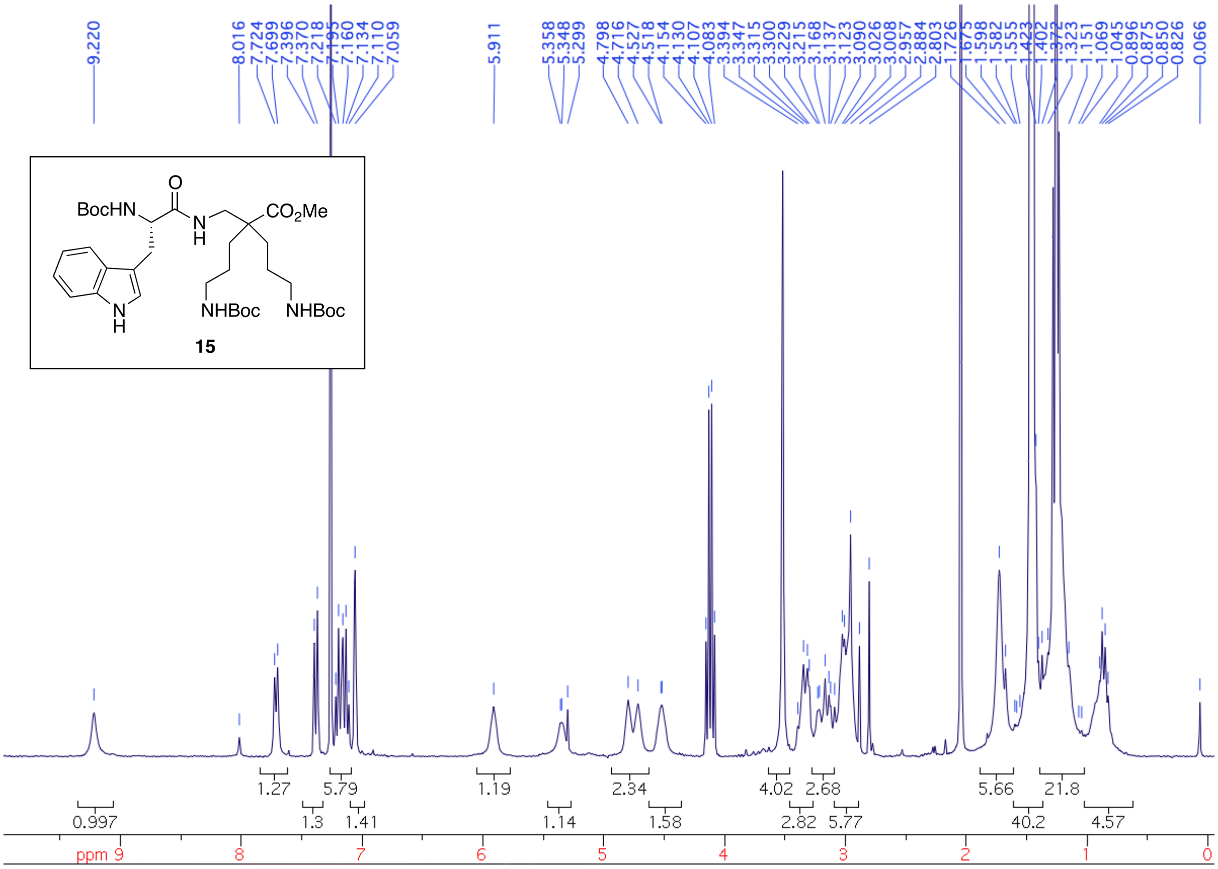

^{1}H NMR spectra of Trp-β^{2,2}*h* bis-Orn-OMe 8, 300MHz, D_2O

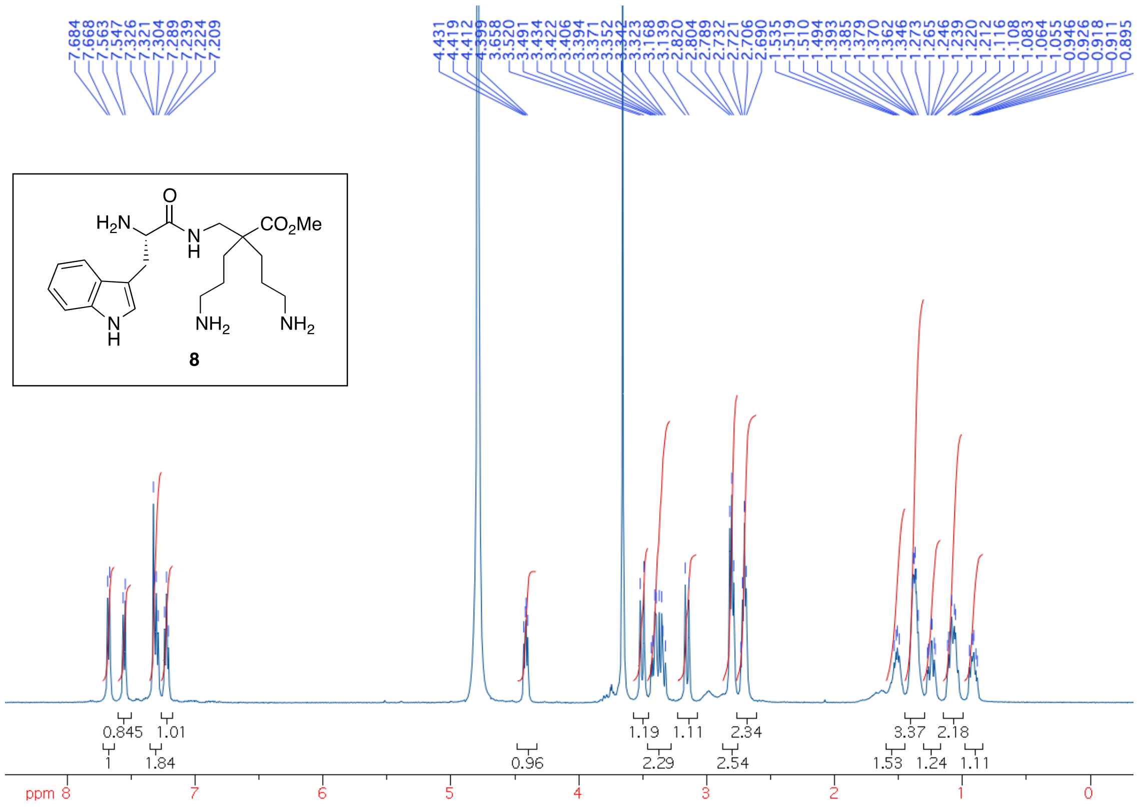

COSY NMR spectra of Trp-β^{2,2}*h* bis-Orn-OMe 8, 300MHz, D_2O

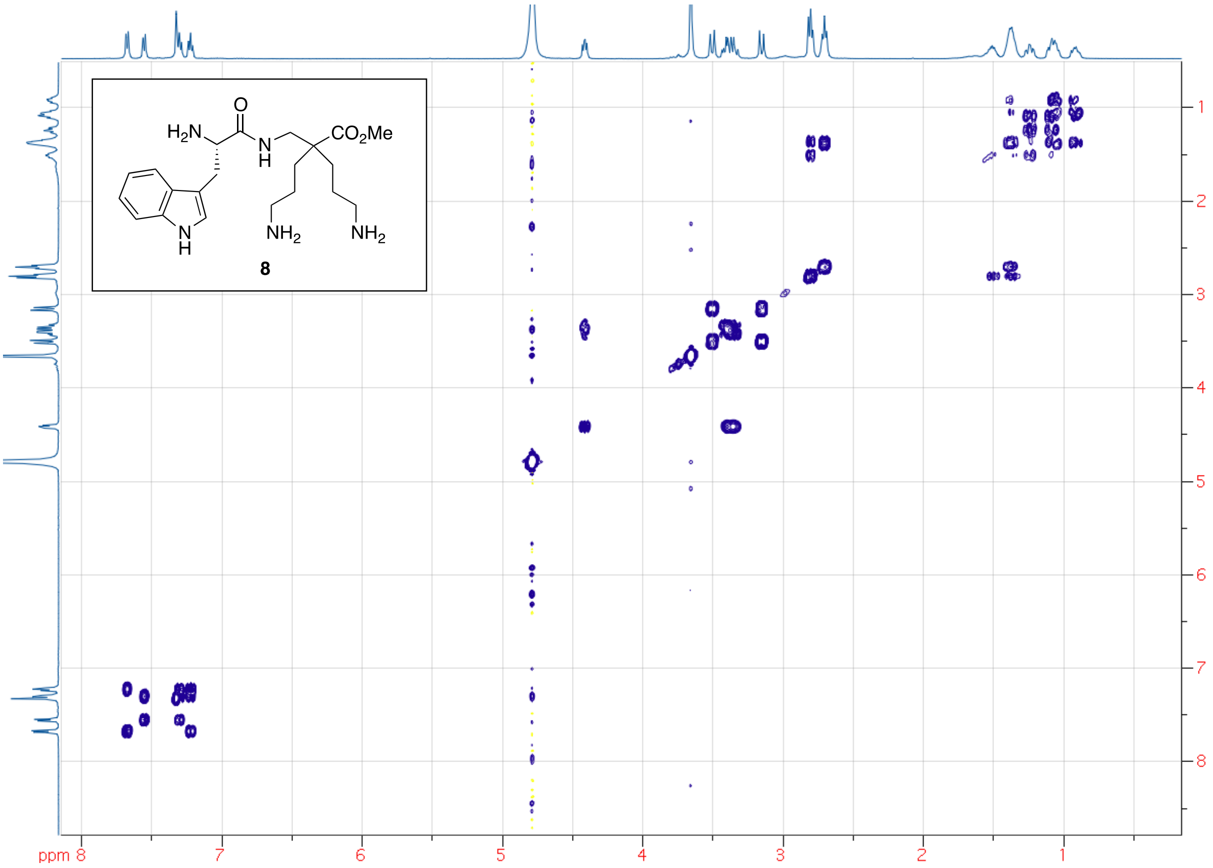

TOCSY NMR spectra of Trp-β^{2,2}*h* bis-Orn-OMe 8, 300MHz, D_2O

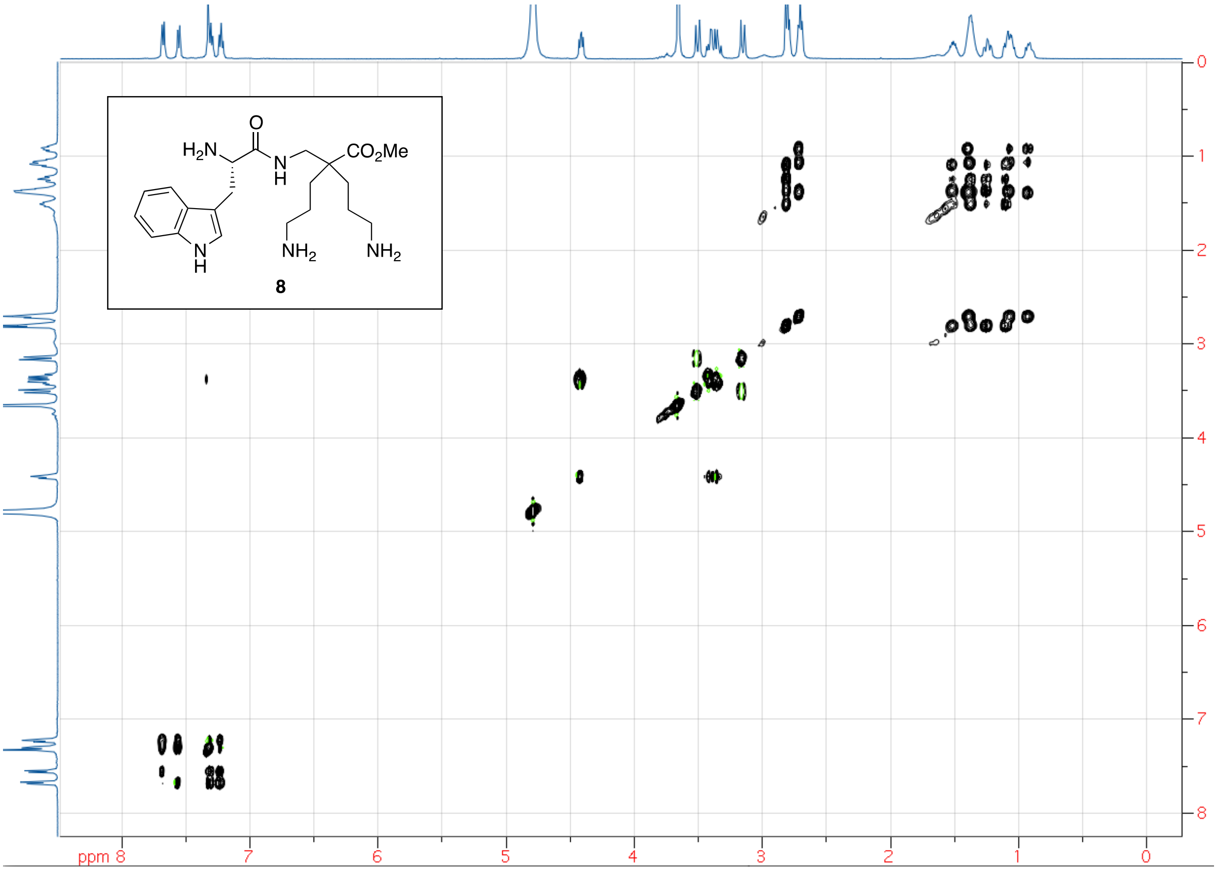

^{1}H NMR spectra of Gua-Trp-$\beta^{2,2}$*h* bis-Arg-OMe 10, 300MHz, D_2O

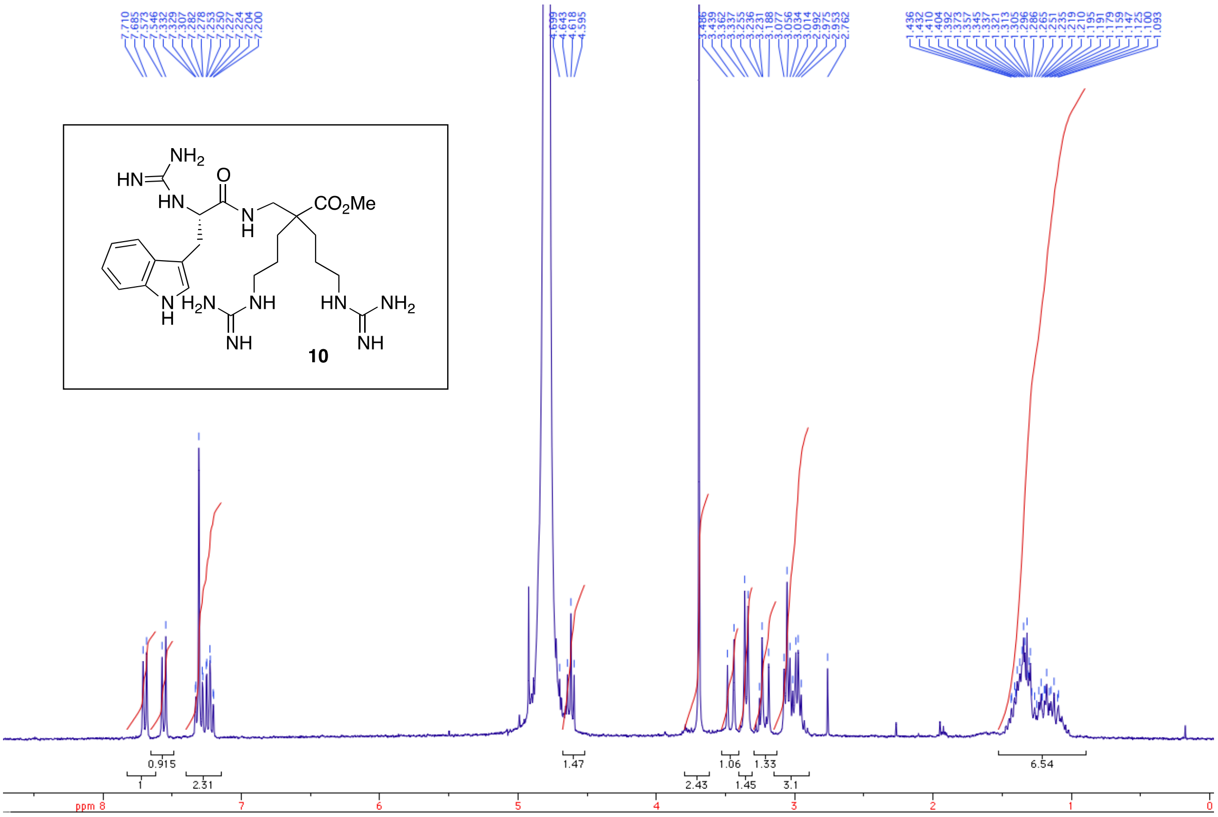

COSY NMR spectra of Gua-Trp-$\beta^{2,2}$*h* bis-Arg-OMe 10, 300MHz, CD_3OD

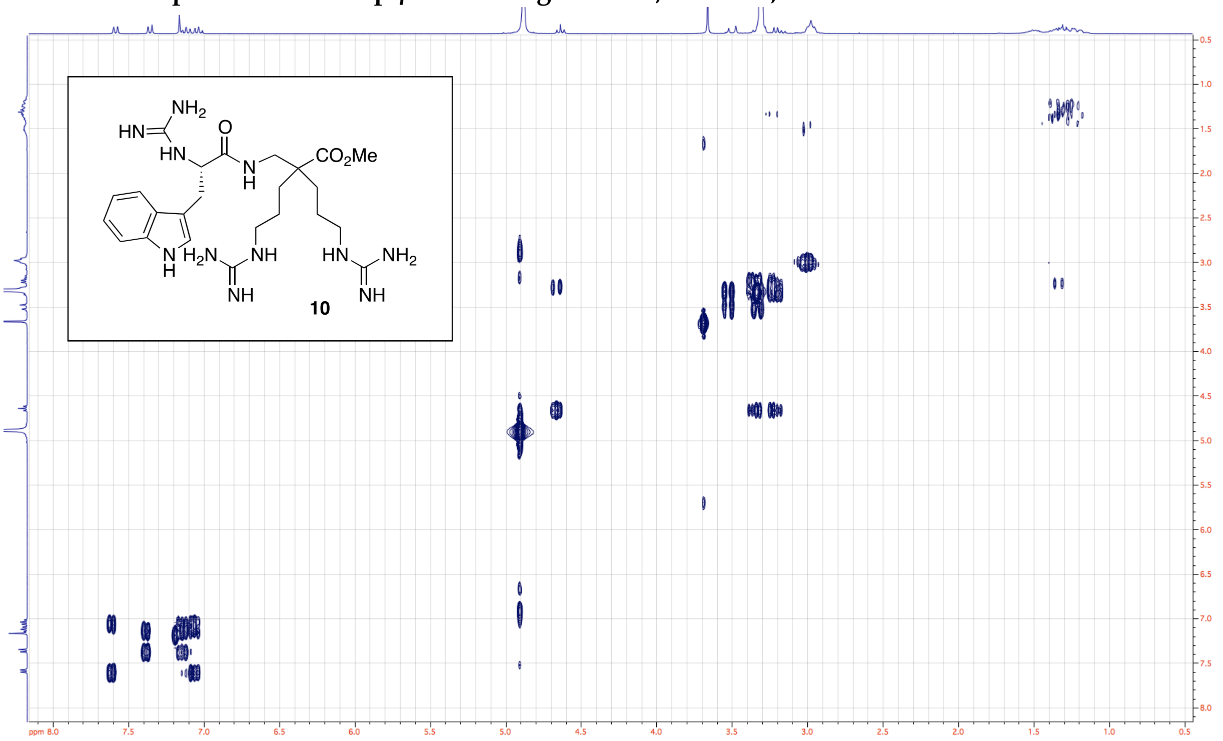

^{1}H NMR spectra of Fmoc-Tbt-β^{2,2} *h* bis-Orn(Boc)$_2$OMe 16, 300 MHz, CD$_3$OD

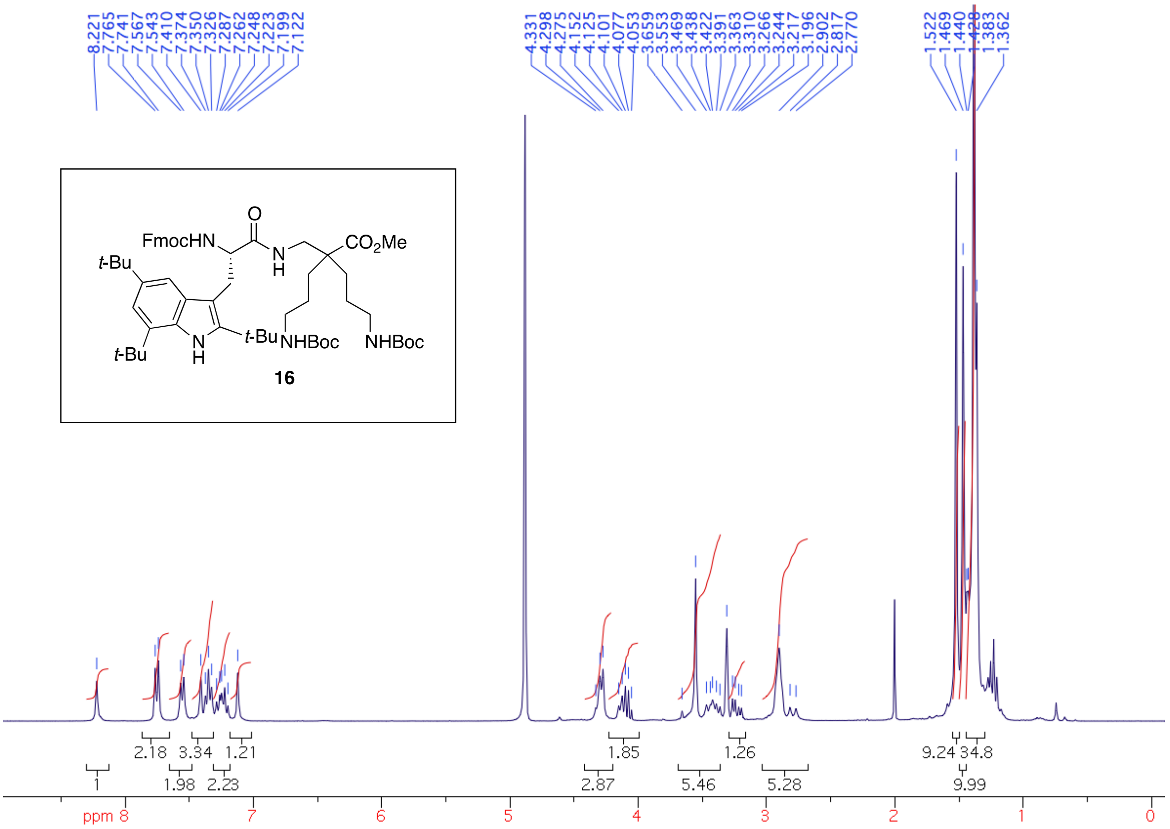

^{13}C NMR spectra of Fmoc-Tbt-β^{2,2} *h* bis-Orn(Boc)$_2$OMe 16, 75 MHz, CD$_3$OD

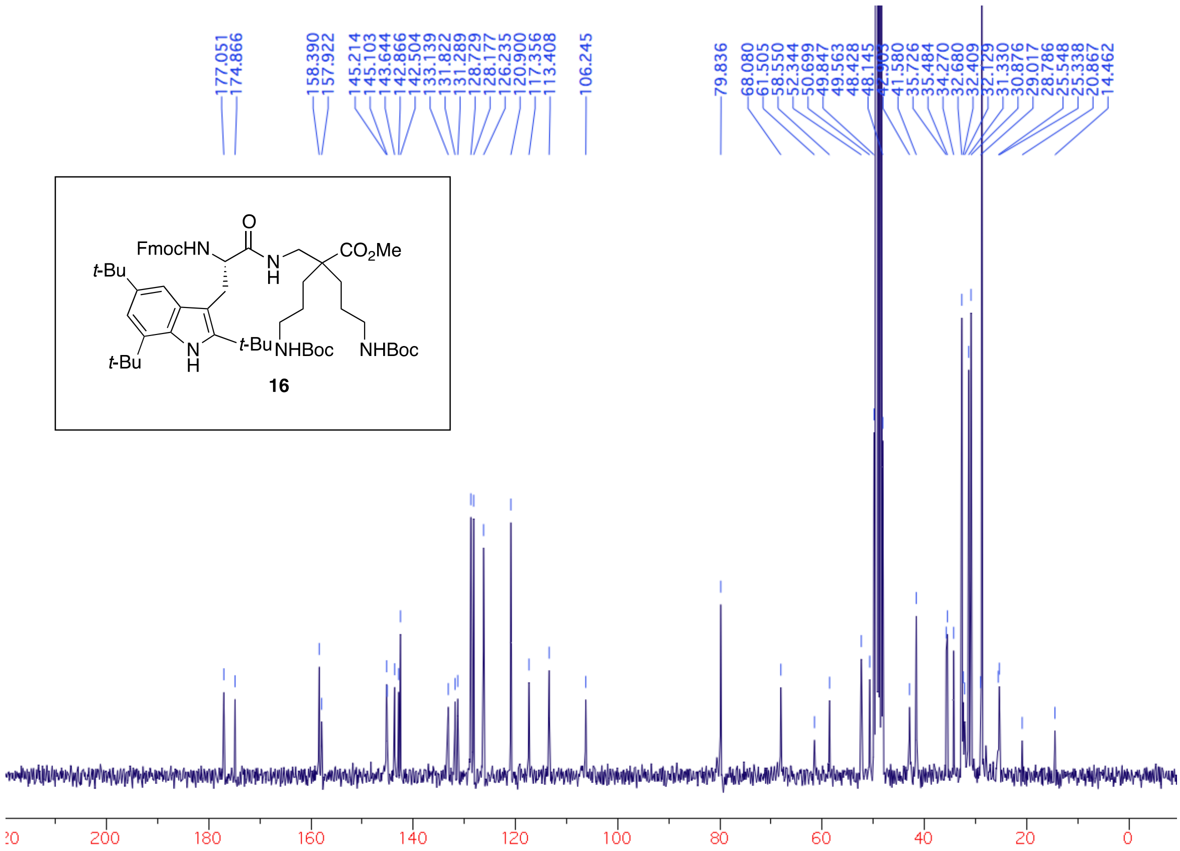

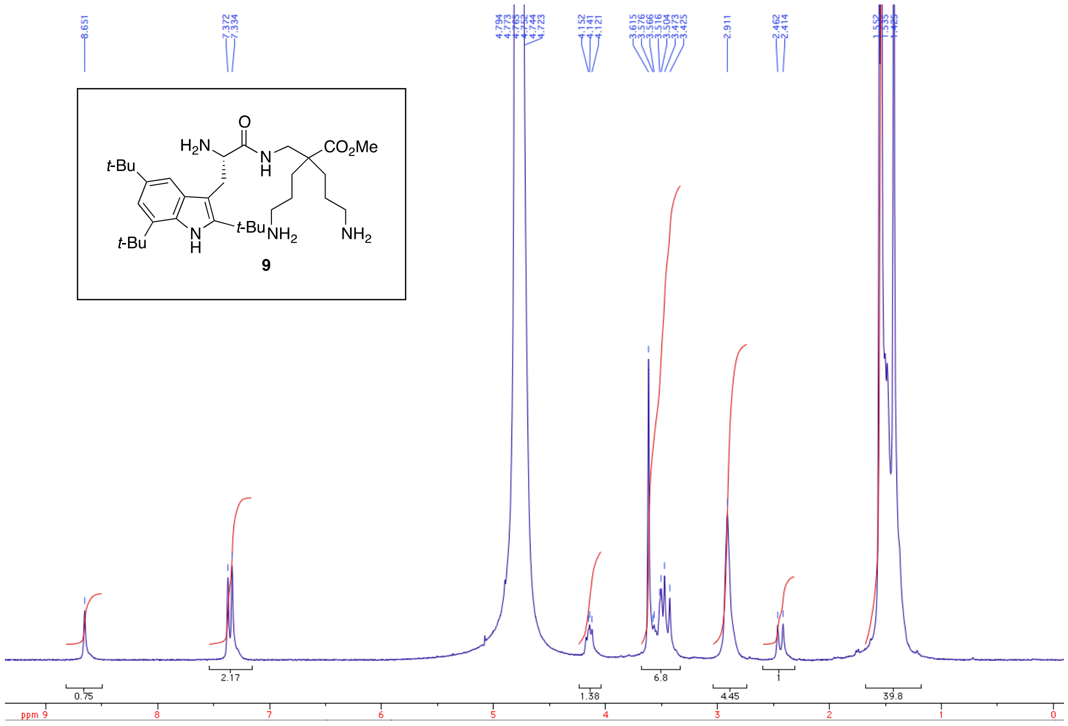

^{1}H NMR spectra of H-Tbt-β[2,2] *h* bis-OrnOMe 9, 300MHz, D2O

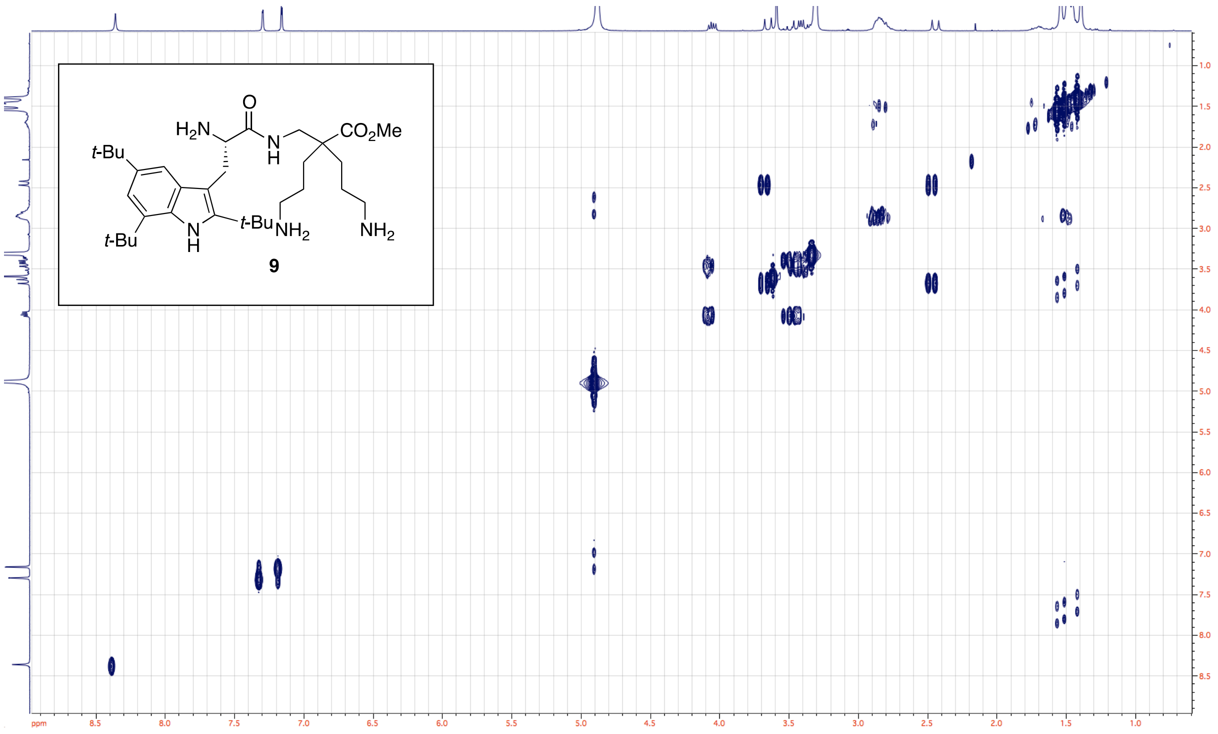

¹H NMR spectra of H-Tbt-β^{2,2}*h* bis-Arg-OMe 11, 500MHz, D_2O

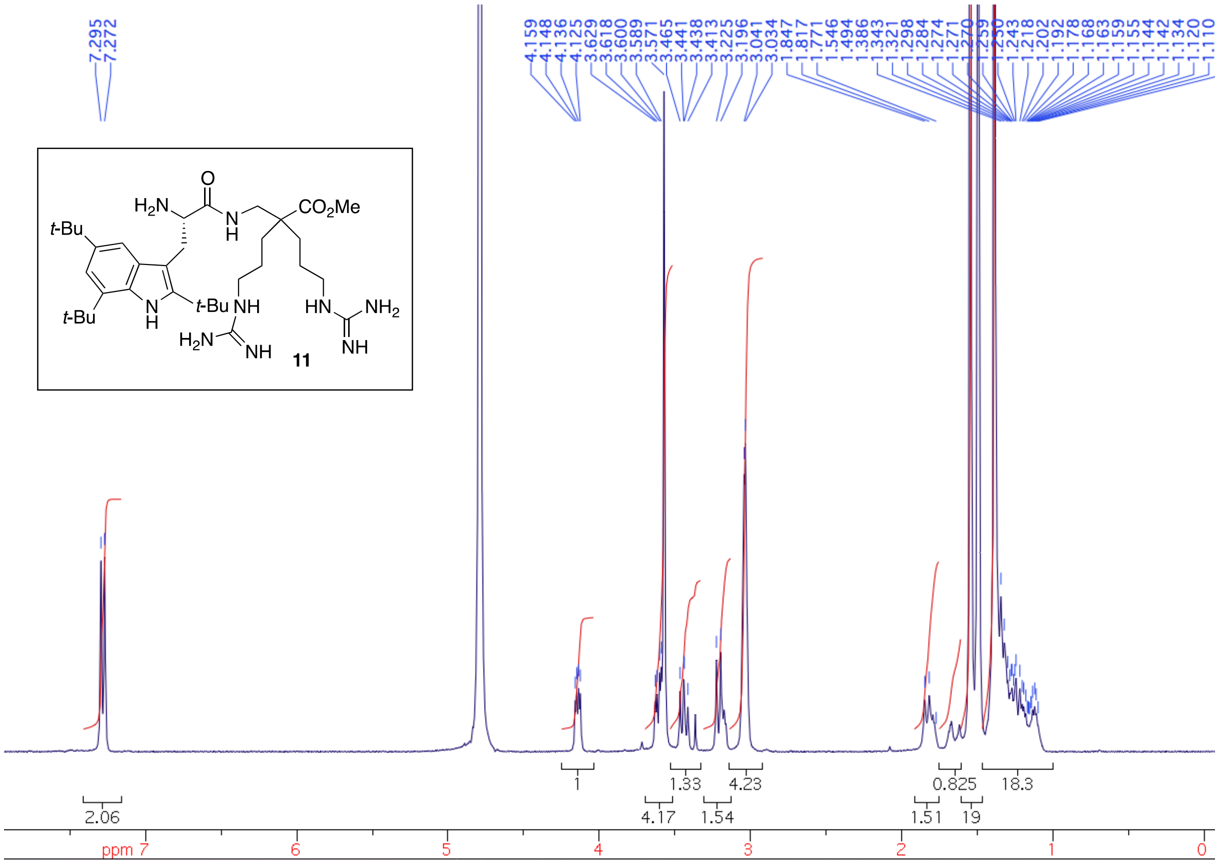

COSY NMR spectra of H-Tbt-β^{2,2}*h* bis-Arg-OMe 11, 500MHz, D_2O

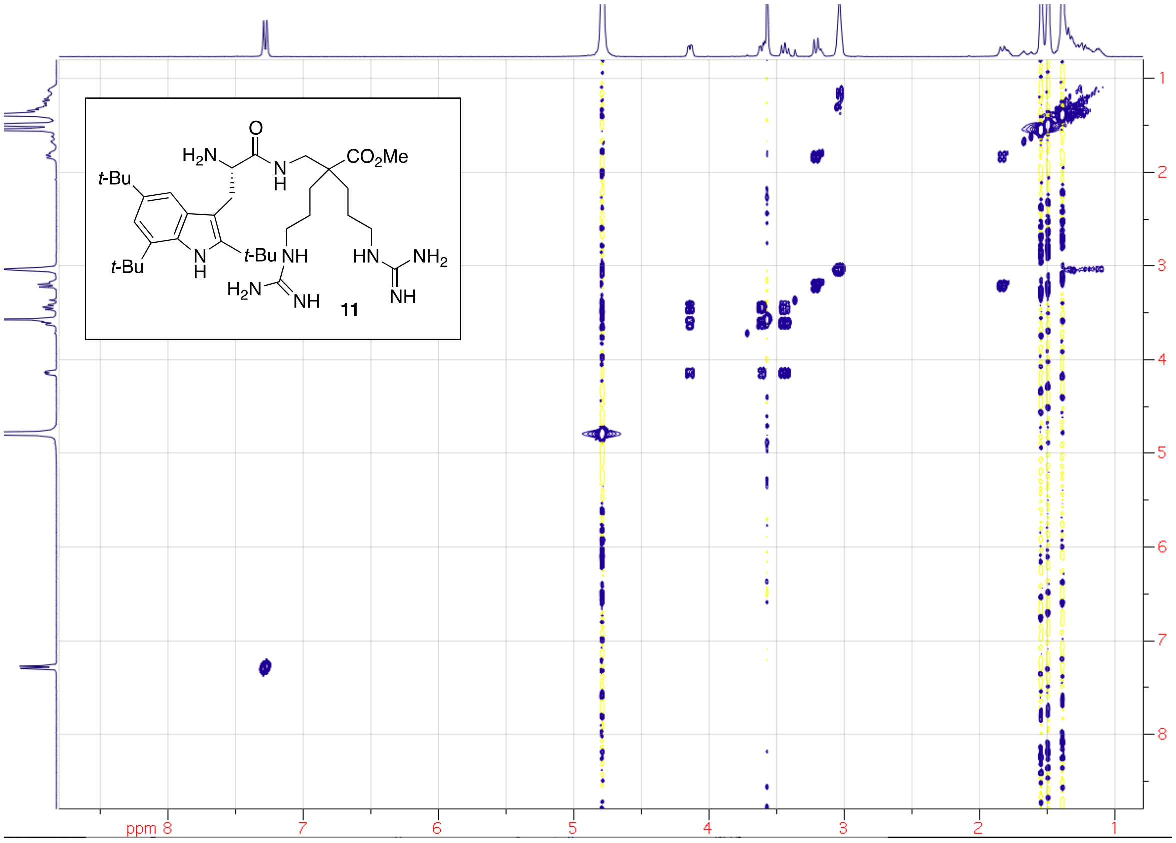

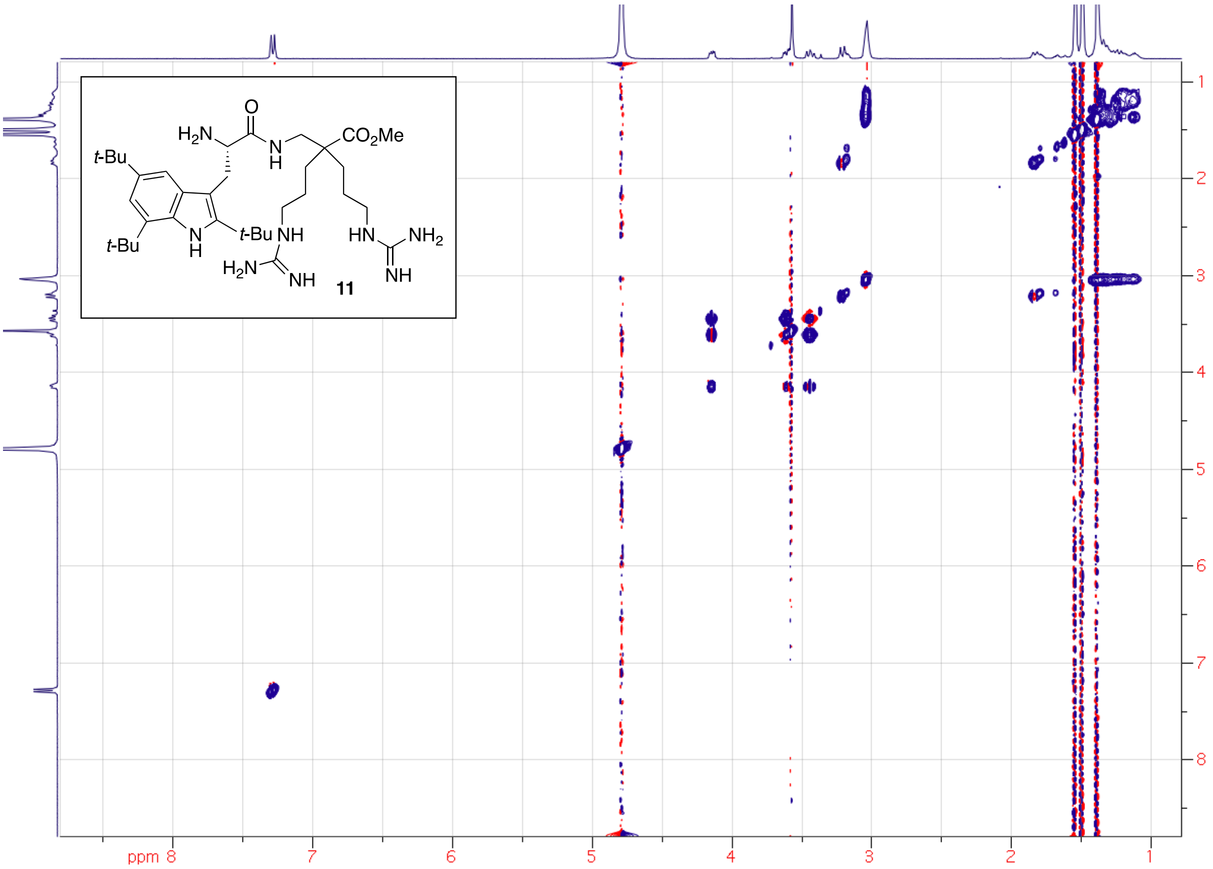

^{1}H NMR spectra of Gdm-Tbt-β[2,2] *h* bis-Arg-OMe 12, 400MHz, MeOD

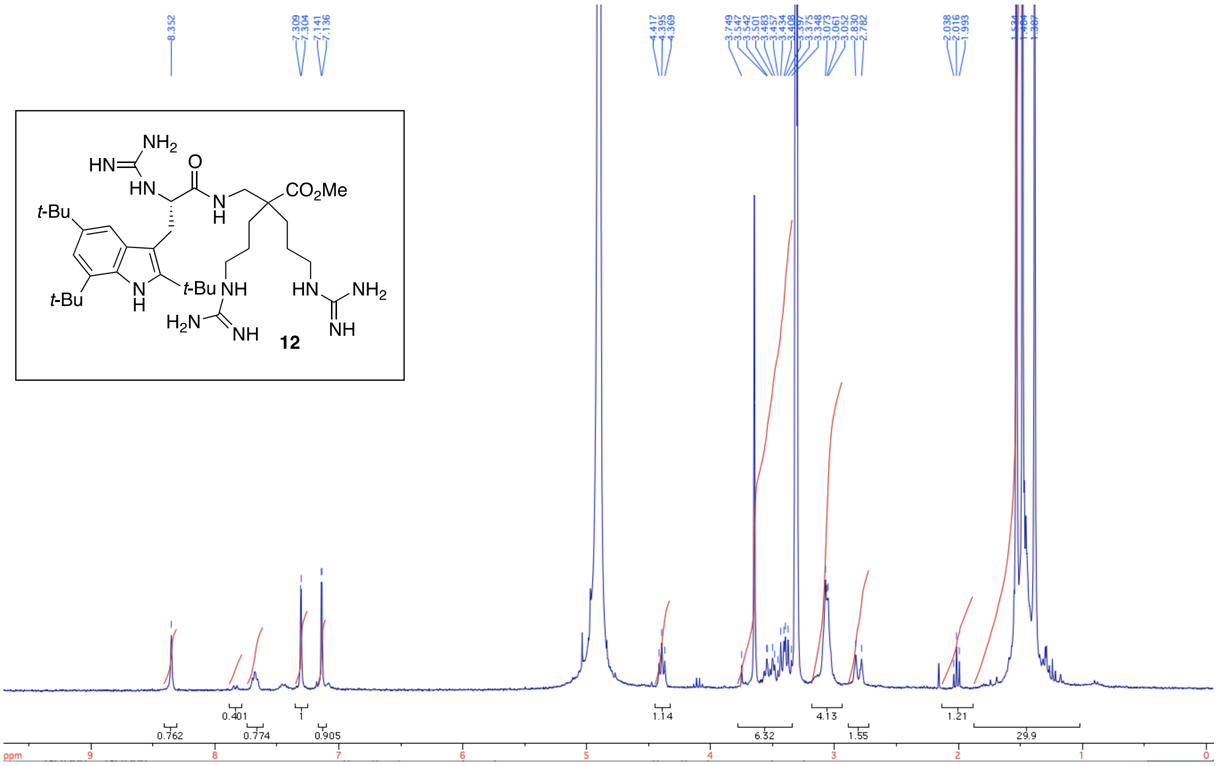

^{1}H NMR spectra of H-β[3,3] *h* bis-Arg-Tbt-OMe 13, 500MHz, D2O

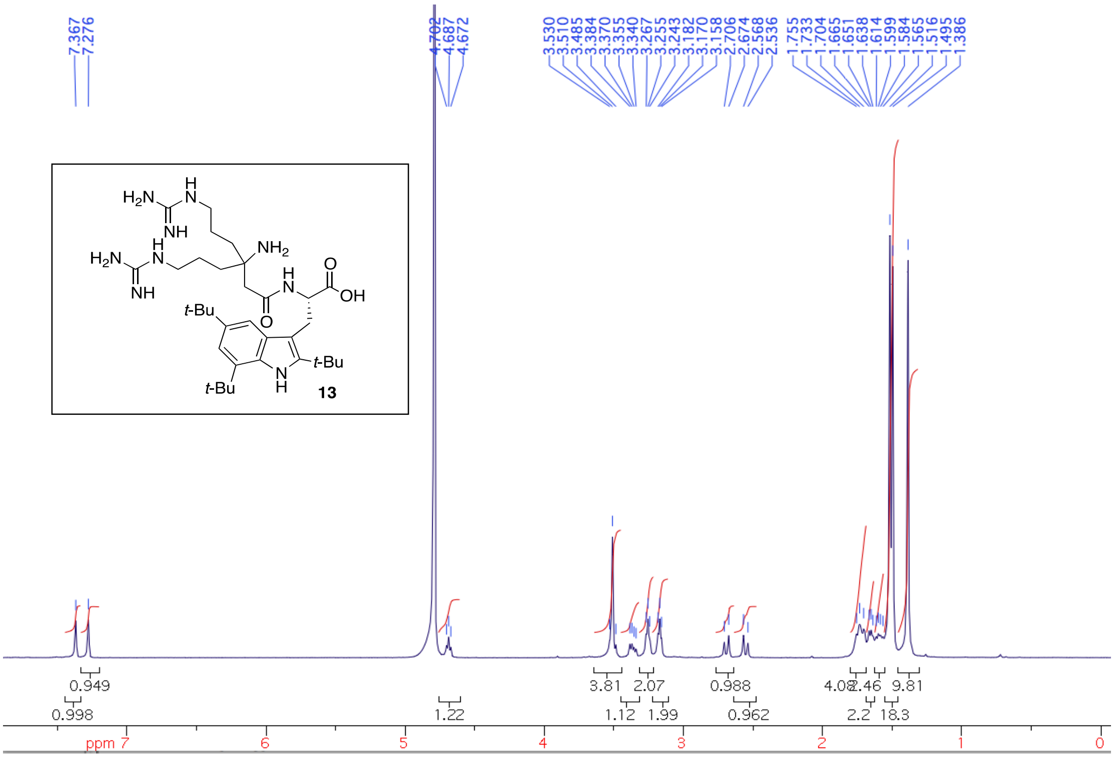

COSY NMR spectra of H-β[3,3] *h* bis-Arg-Tbt-OMe 13, 500MHz, D2O

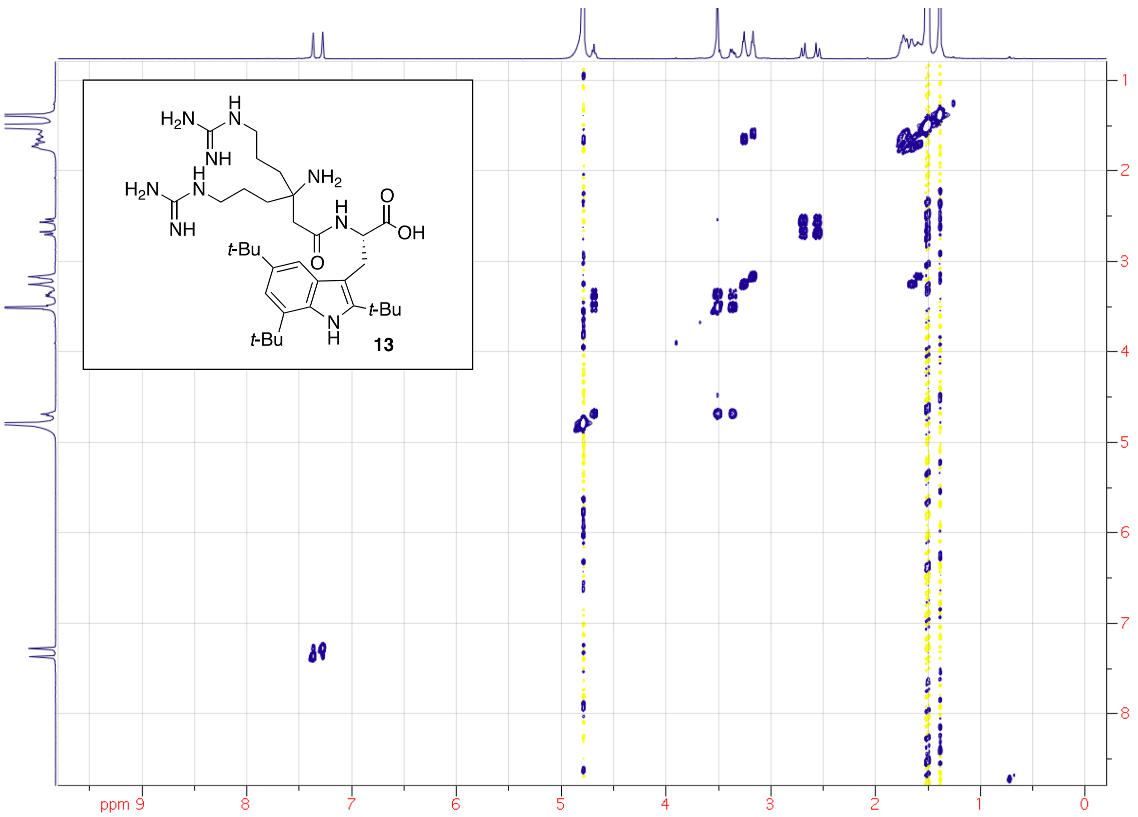

TOCSY NMR spectra of H-β^{3,3} *h* bis-Arg-Tbt-OMe 13, 500MHz, D$_2$O

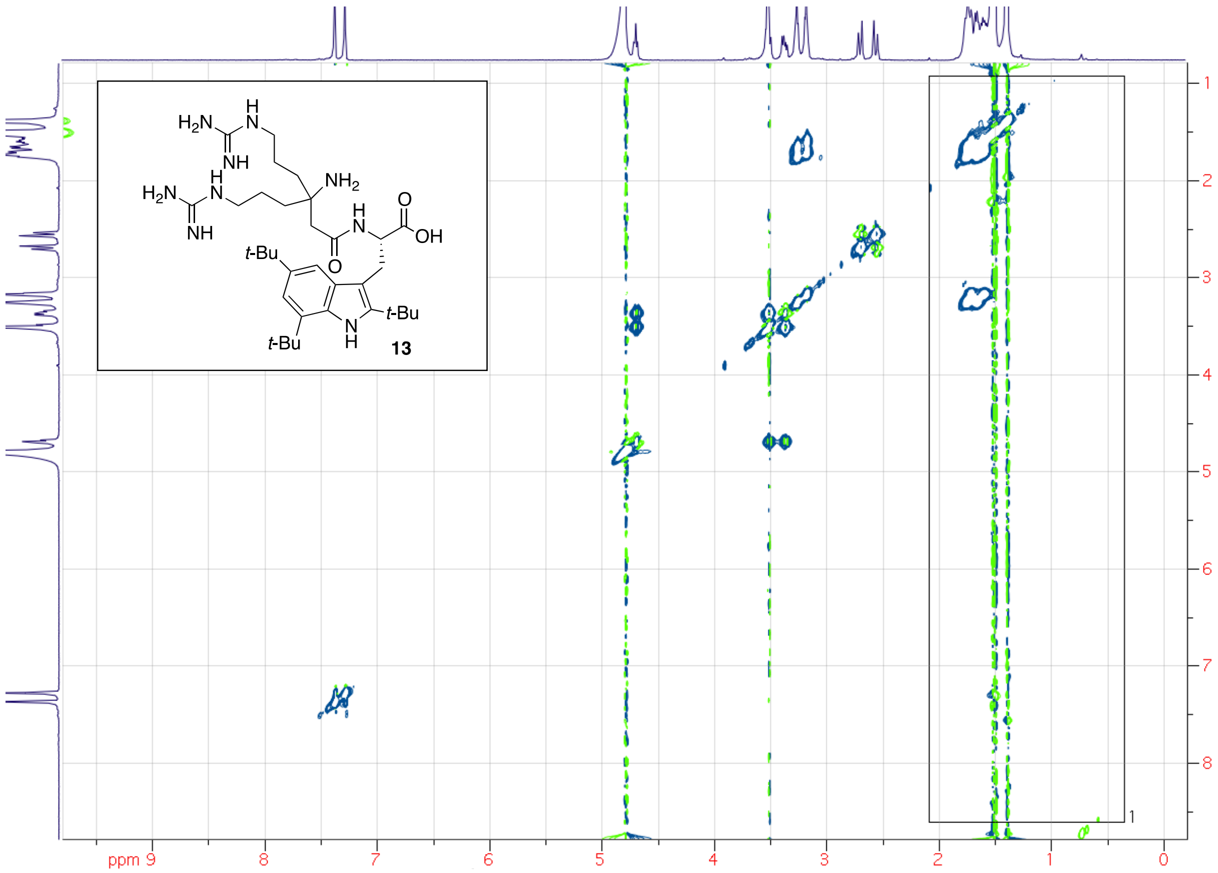

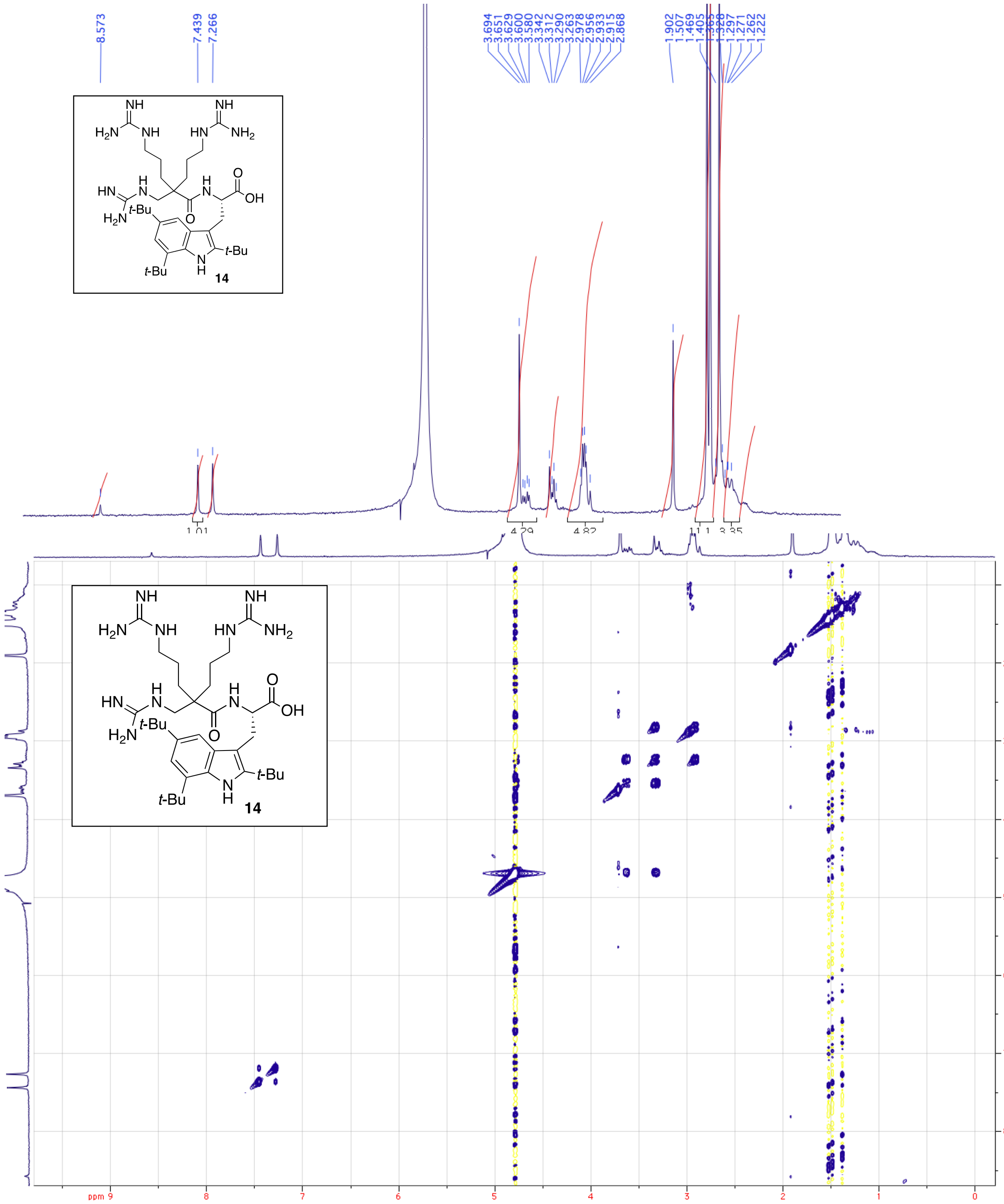
8.573
7.439
7.266
3.694
3.651
3.629
3.600
3.580
3.342
3.312
3.290
3.263
2.978
2.956
2.933
2.915
2.868
1.902
1.507
1.469
1.405
1.297
1.271
1.262
1.222
1.01
4.29
4.82
3.35
t-Bu
14
ppm

TOCSY NMR spectra of Gua-β^2,2^ *h* bis-Arg-Tbt-OMe 14, 500MHz, D₂O

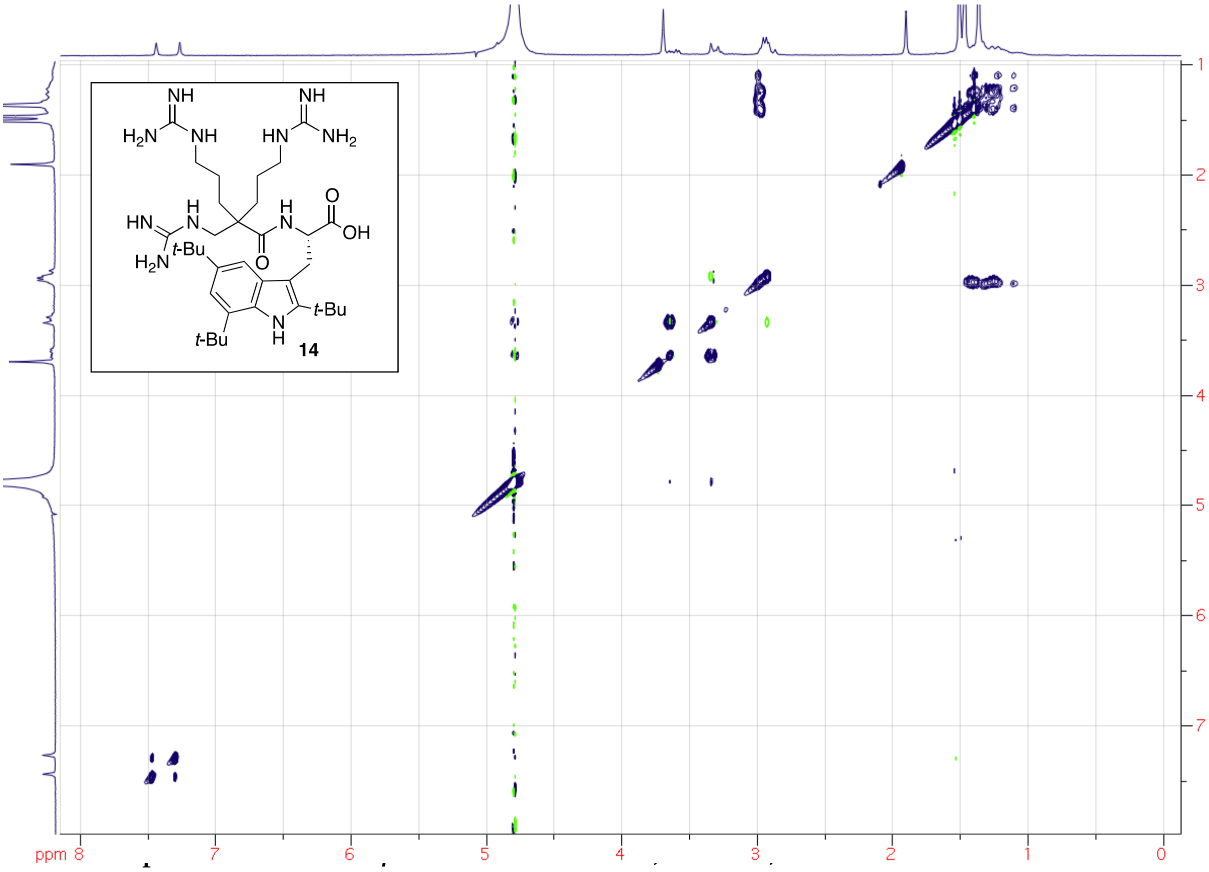

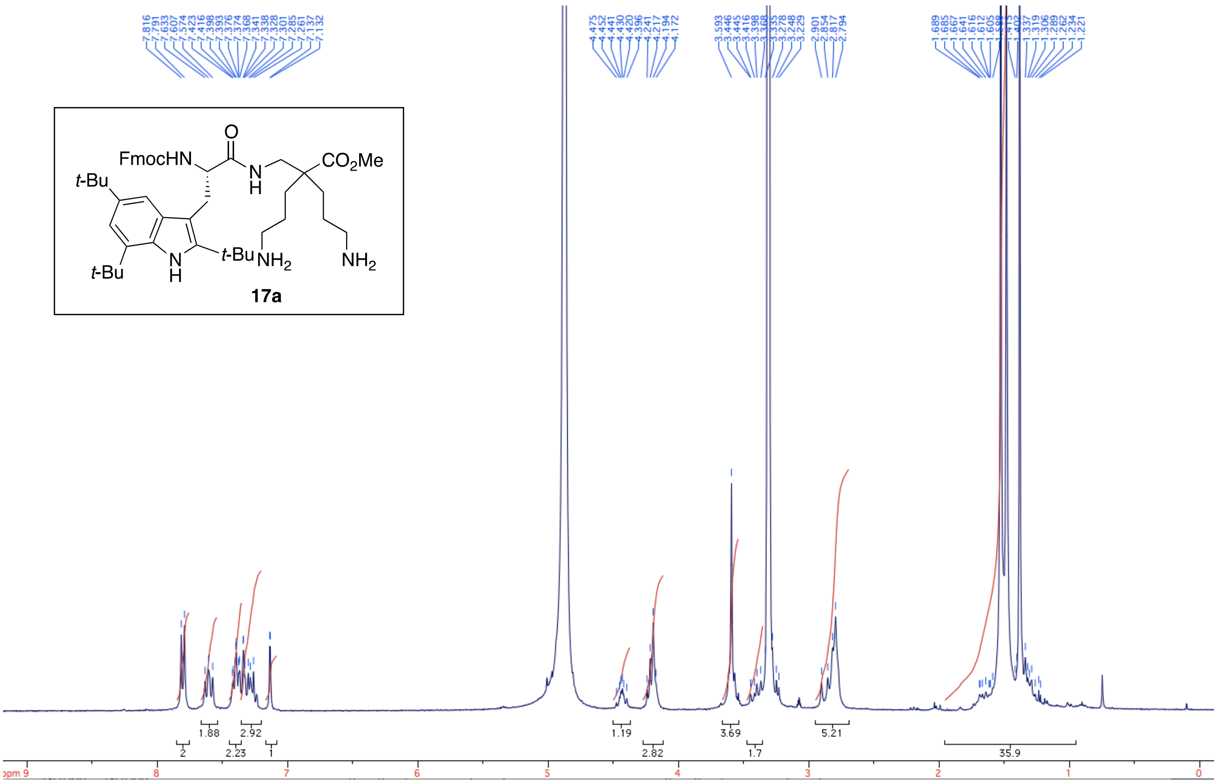

¹H NMR spectra of Fmoc-Tbt-β^2,2^ *h* bis-Arg-OMe 17b, 300MHz, CD₃OD

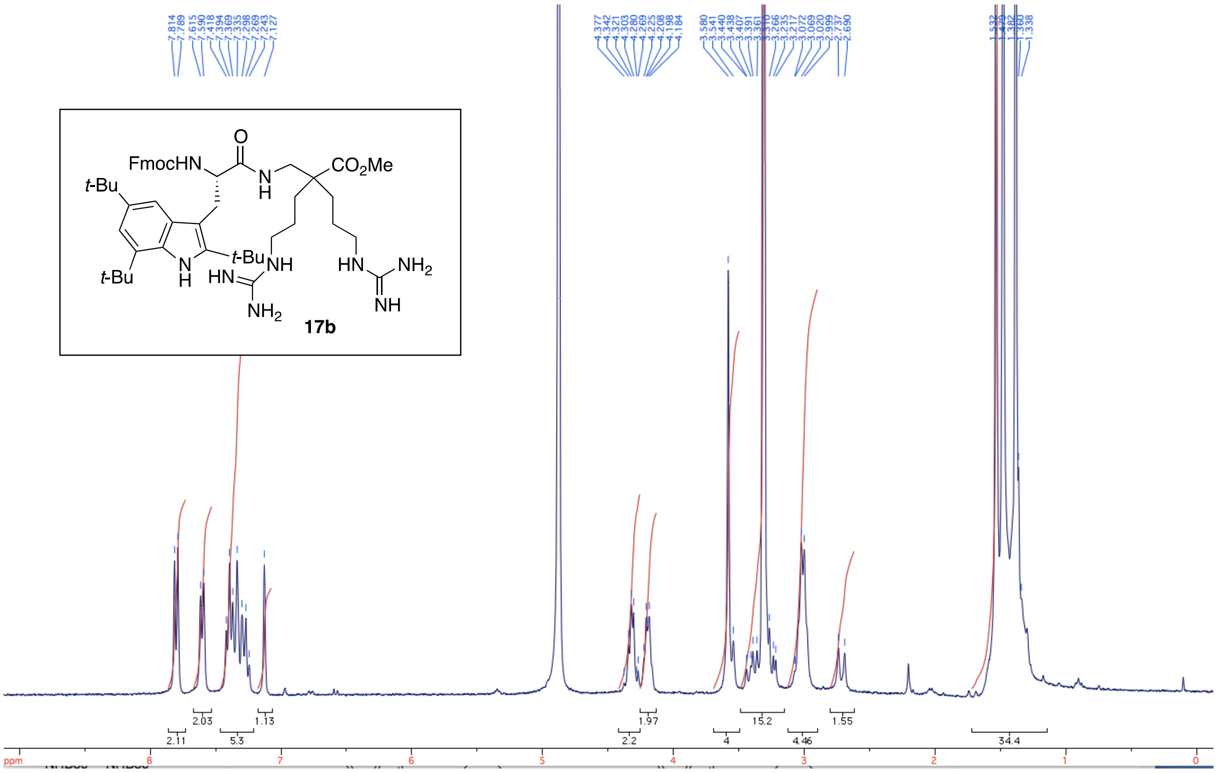

^{1}H NMR spectra of Fluo-Tbt-β^{2,2}*h* bis-Orn-OMe 18, 300MHz, CD$_3$OD

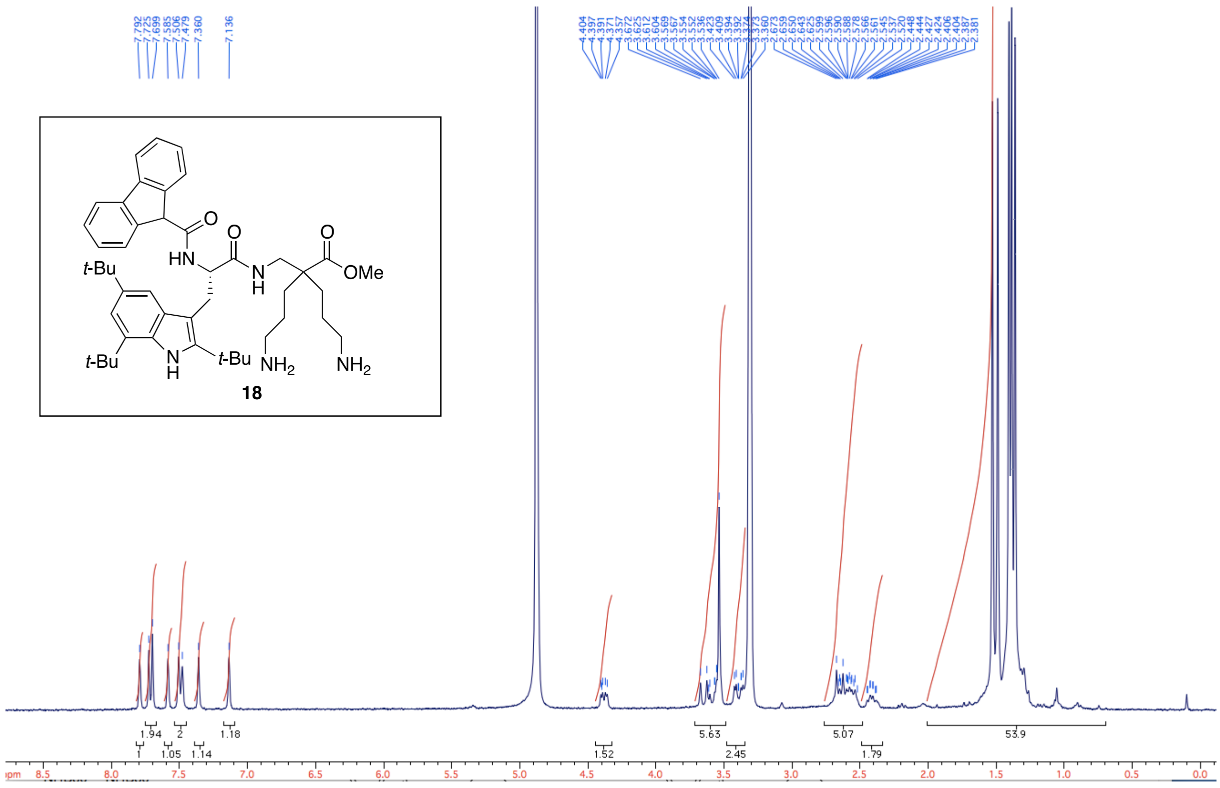

^{1}H NMR spectra of Np-Tbt-$\beta^{2,2}$*h* bis-Orn-OMe 19, 300MHz, CD_3OD

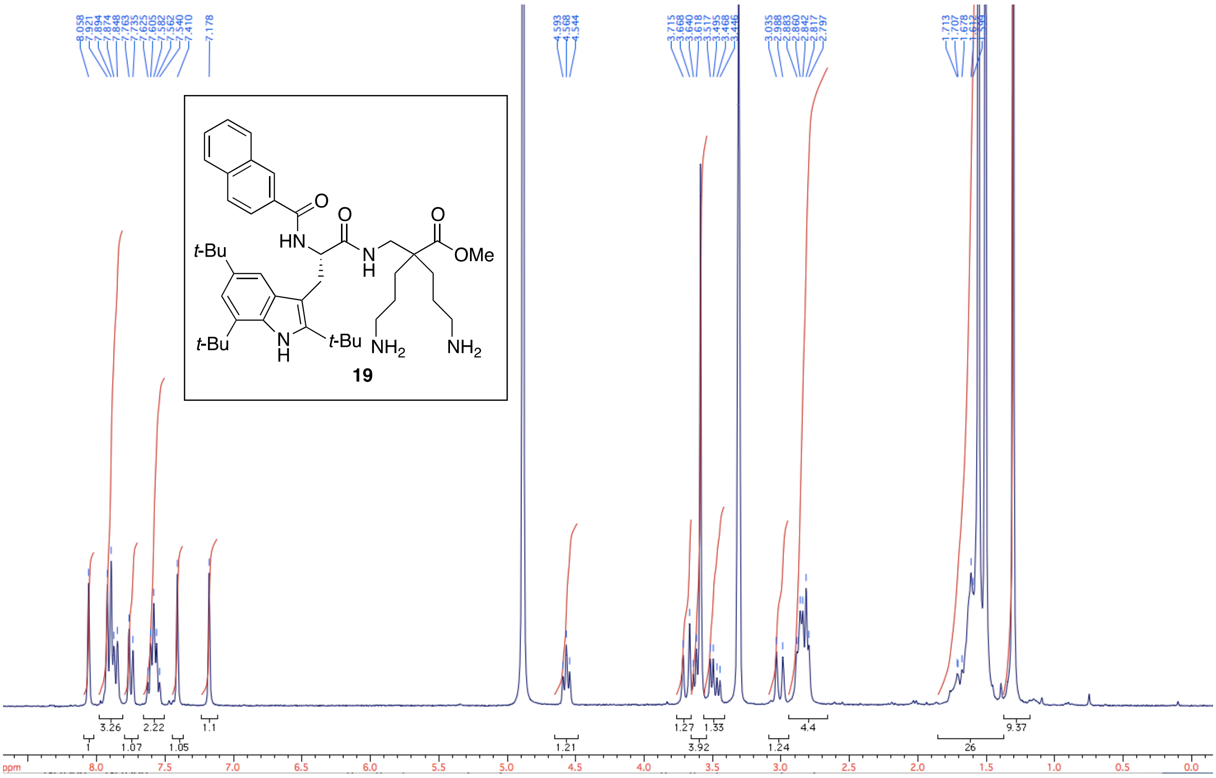

^{1}H NMR spectra of H-$\beta^{2,2}$*h* bis-Orn(Boc)$_2$OBn 27, 300 MHz, CD_3OD

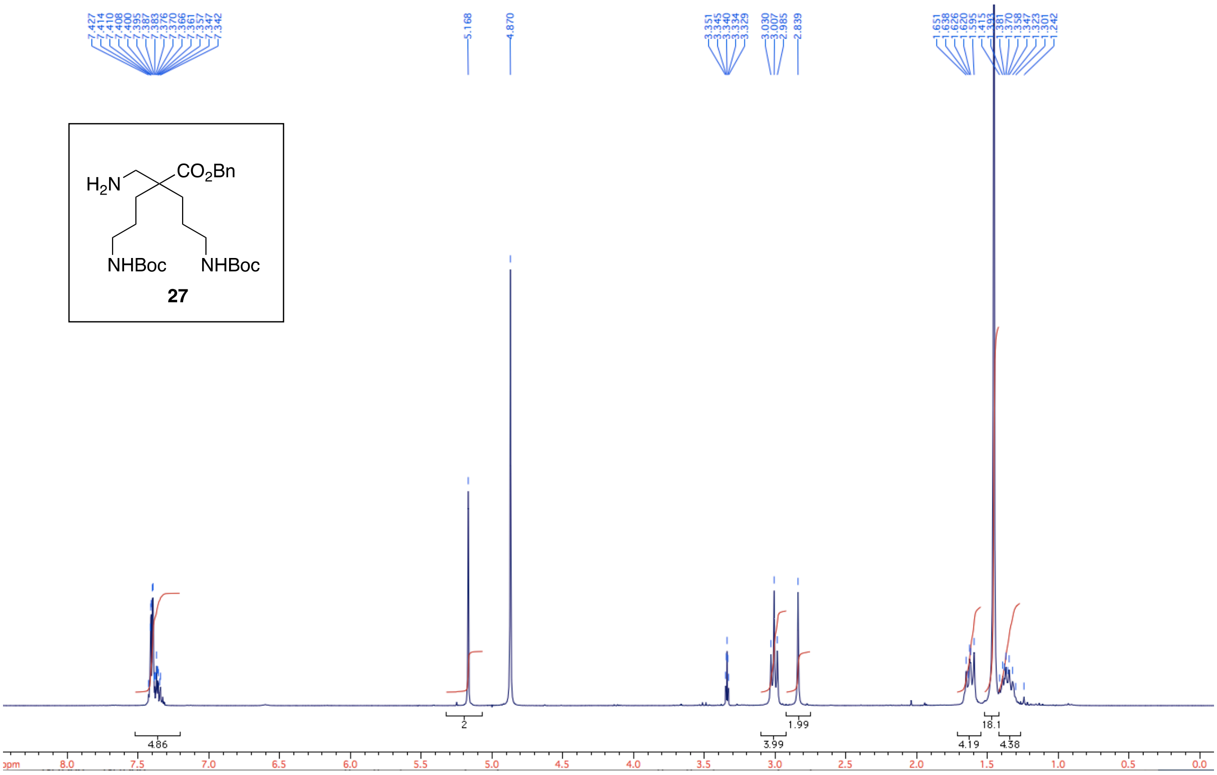

^{13}C NMR spectra of H-$\beta^{2,2}$*h* bis-Orn(Boc)$_2$OBn 27, 300 MHz, CD_3OD

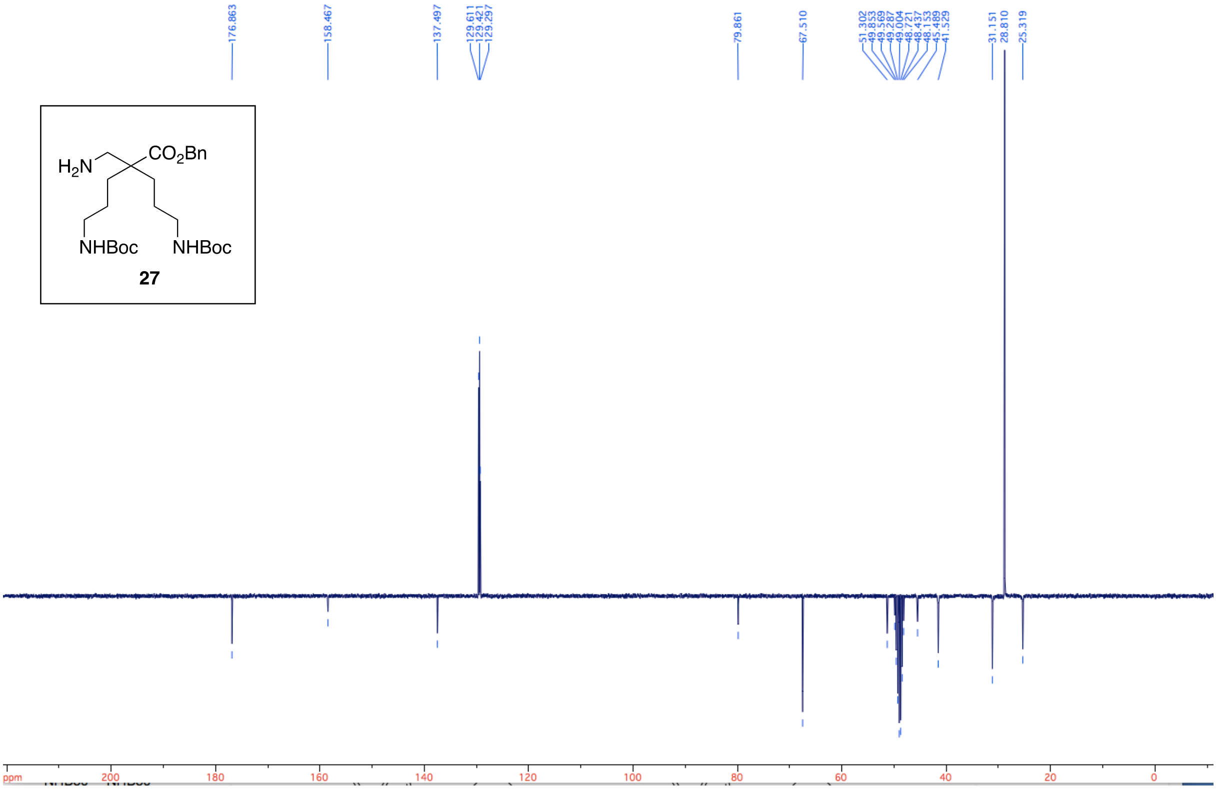

^{1}H NMR spectra of Fmoc-Tbt-$\beta^{2,2}$*h* bis-Orn(Boc)$_2$-OBn 28, 300MHz, CD_3OD

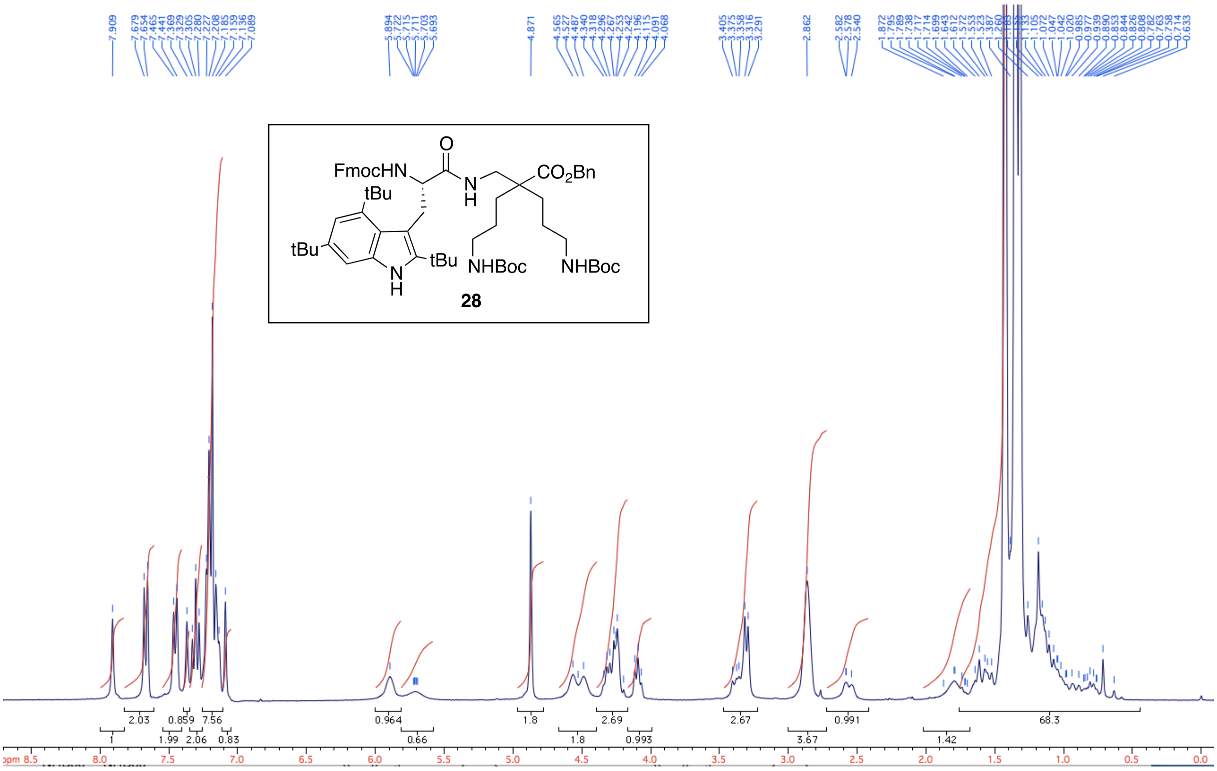

^{1}H NMR spectra of H-Tbt-β^{2,2} *h* bis-Arg-OBn 20, 300MHz, CD_3OD

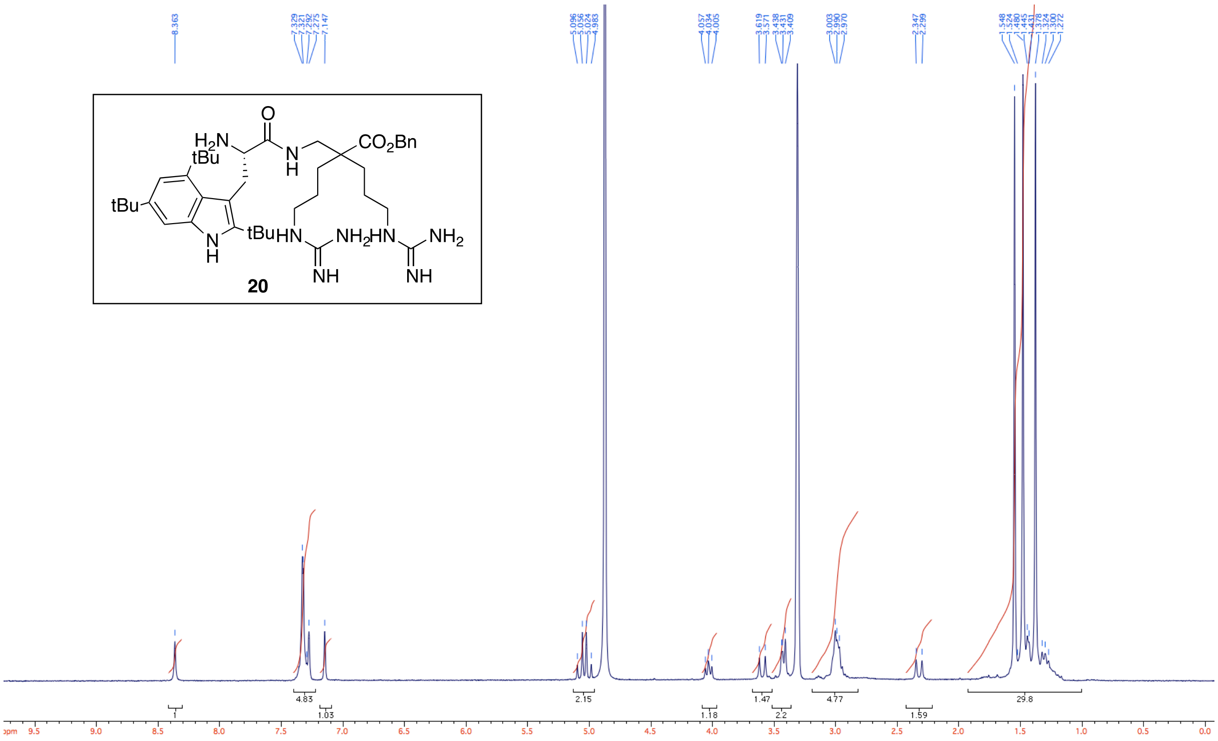

COSY NMR spectra of H-Tbt-β^{2,2} *h* bis-Arg-OBn 20, 300MHz, CD_3OD

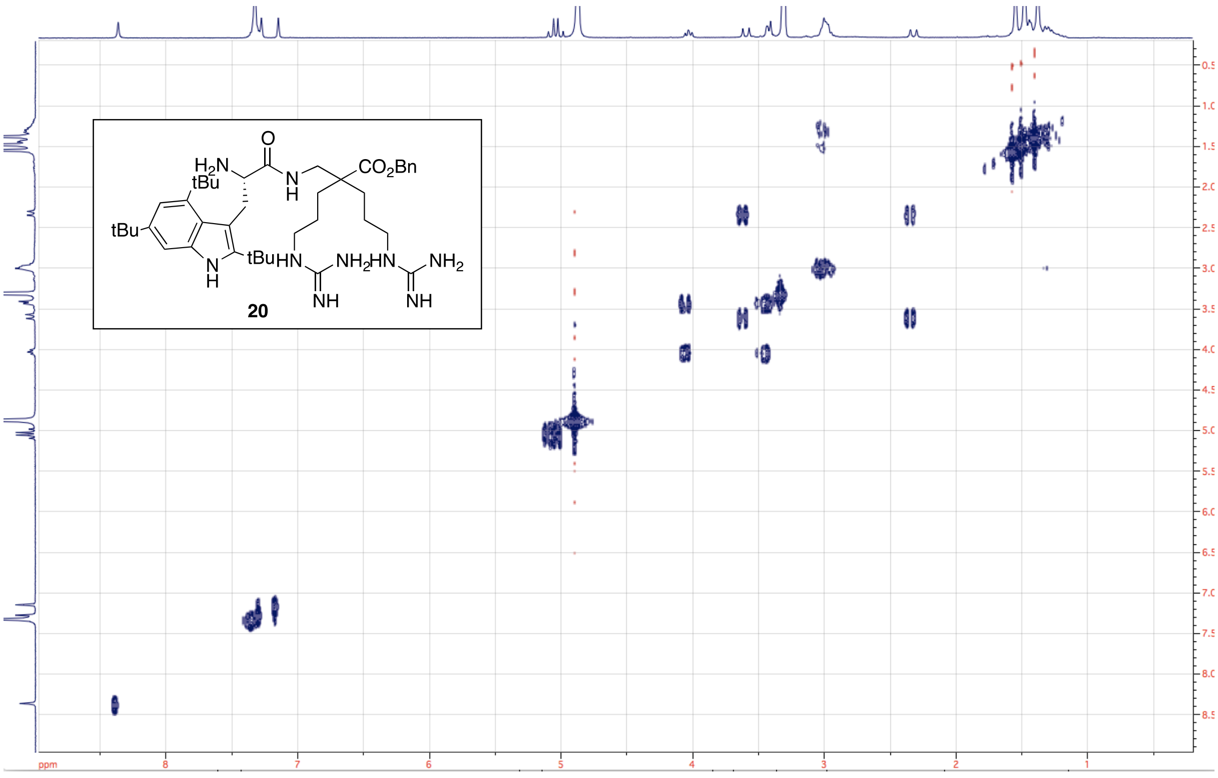

^{1}H NMR spectra of Fmoc-β^{2,2}*h* bis-Orn(Boc)$_2$NHBn, 300 MHz, CD_3OD

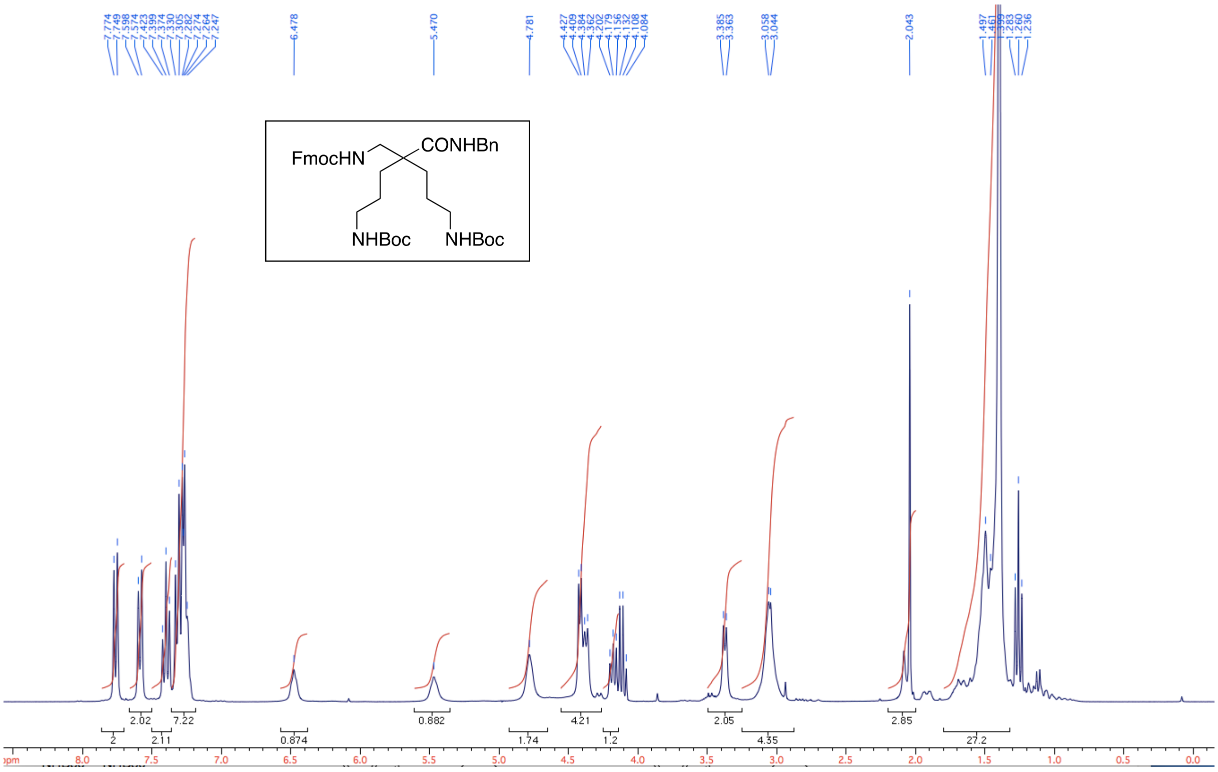

^{13}C NMR spectra of Fmoc-β^{2,2}*h* bis-Orn(Boc)$_2$NHBn, 300 MHz, CD_3OD

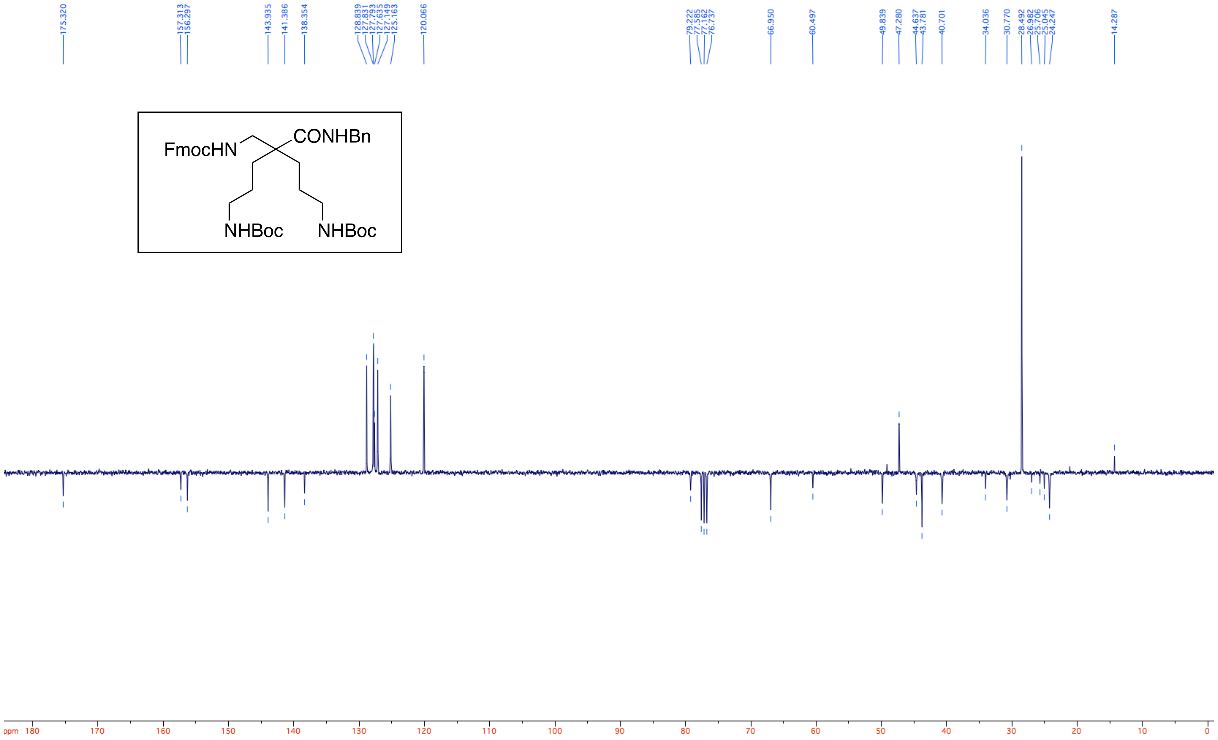

^{1}H NMR spectra of H-β^{2,2} *h* bis-Orn(Boc)$_2$NHBn 29, 300 MHz, CD_3OD

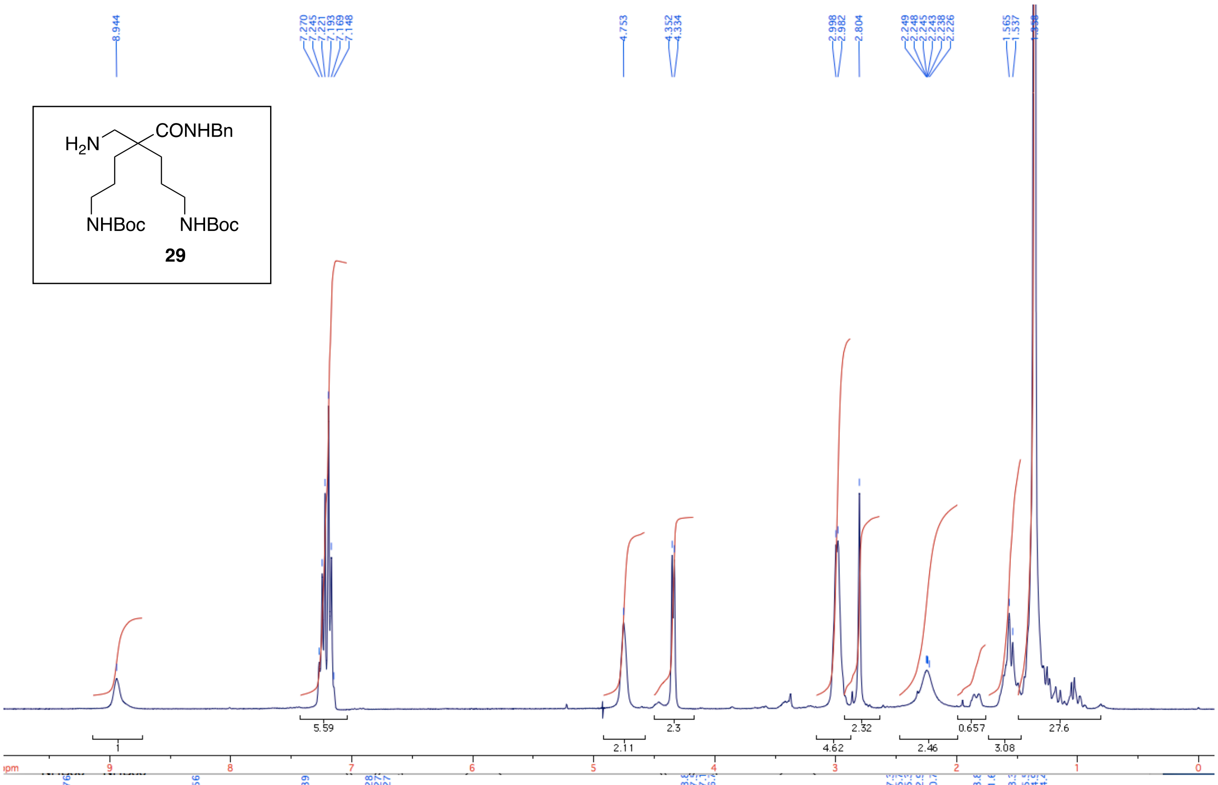

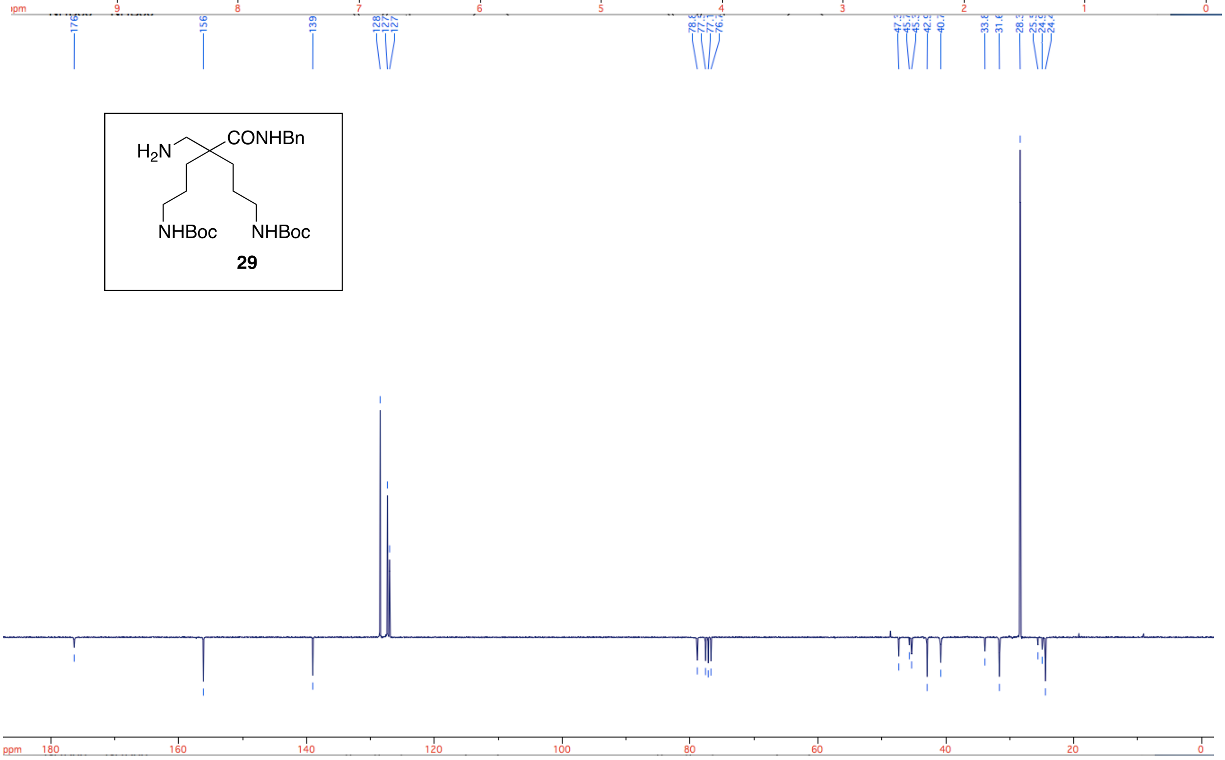

^{1}H NMR spectra of Fmoc-Tbt-$\beta^{2,2}$ *h* bis-Orn(Boc)$_2$-NHBn 30, 300MHz, CD_3OD

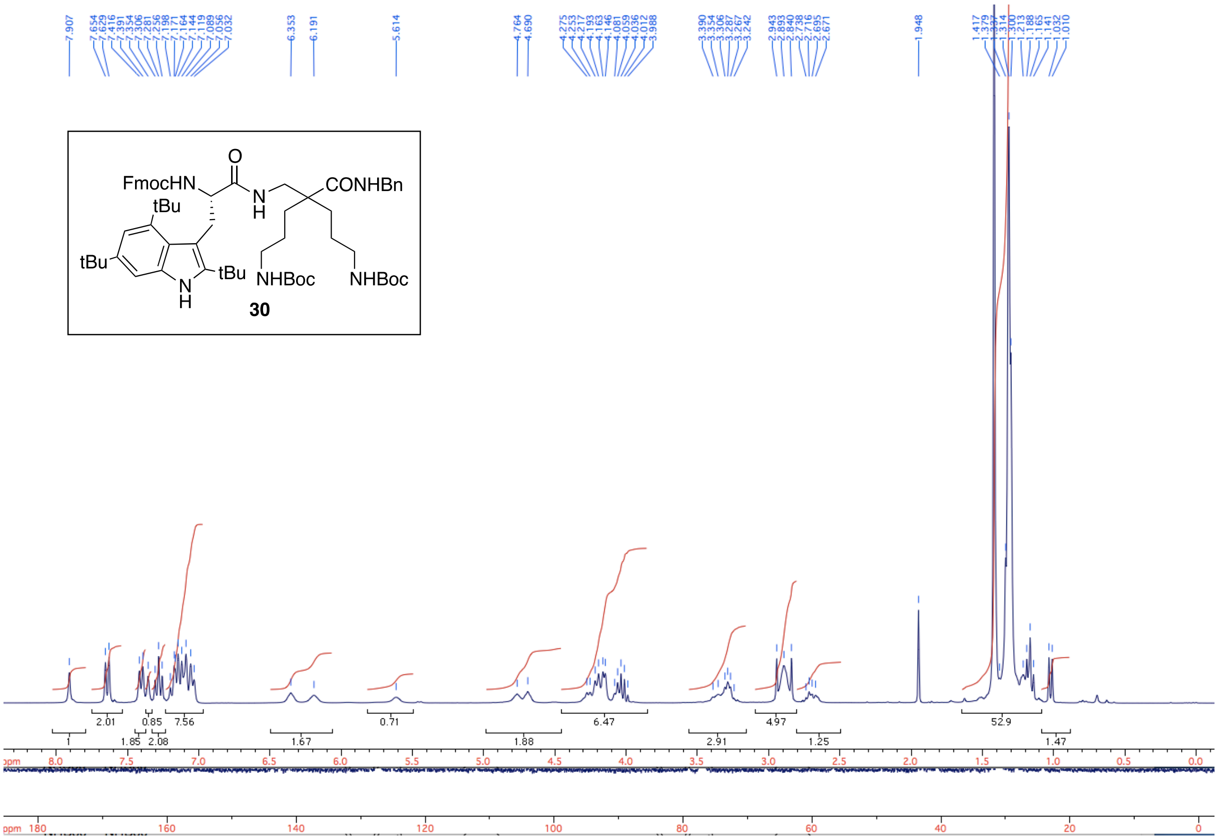

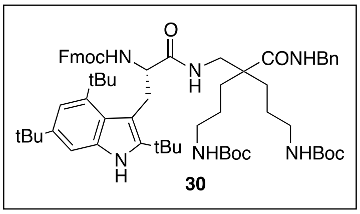

^{1}H NMR spectra of H-Tbt-β[2,2] *h* bis-Arg-NHBn 21, 300MHz, CD_3OD

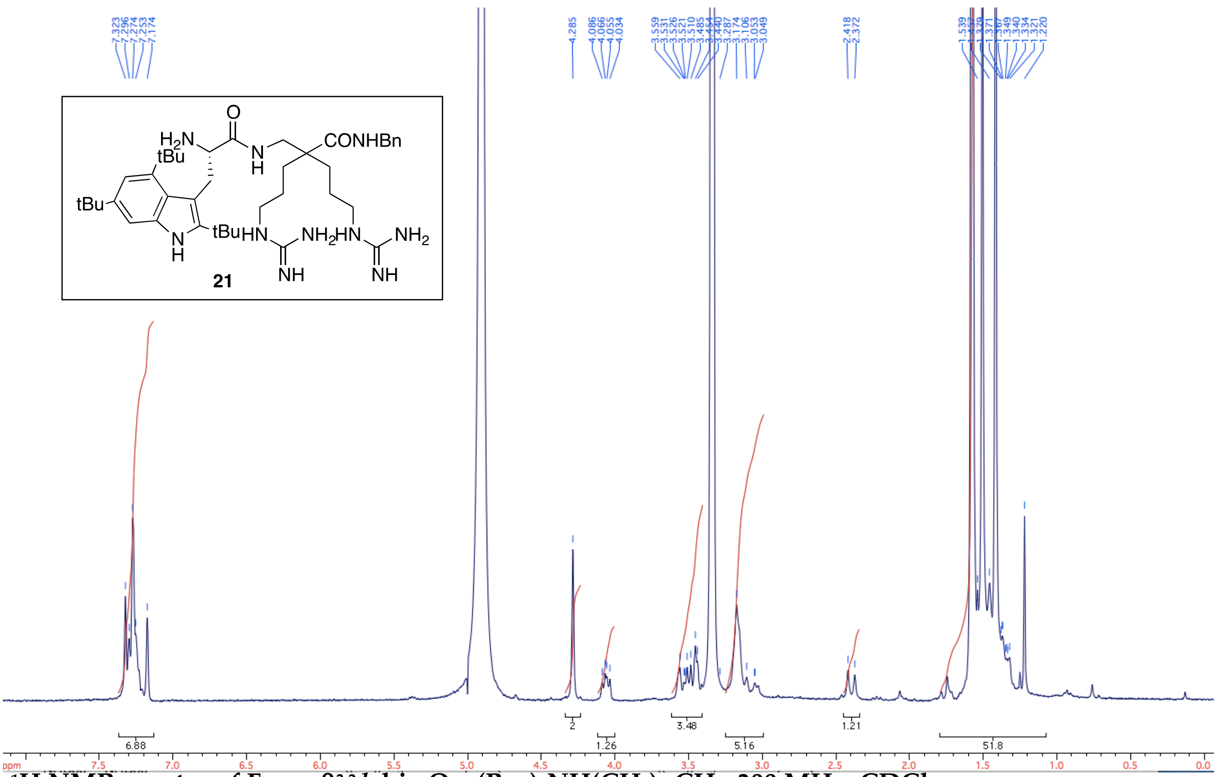

^{1}H NMR spectra of Fmoc-β[2,2] *h* bis-Orn(Boc)$_2$NH$(CH_2)_{13}CH_3$, 300 MHz, $CDCl_3$

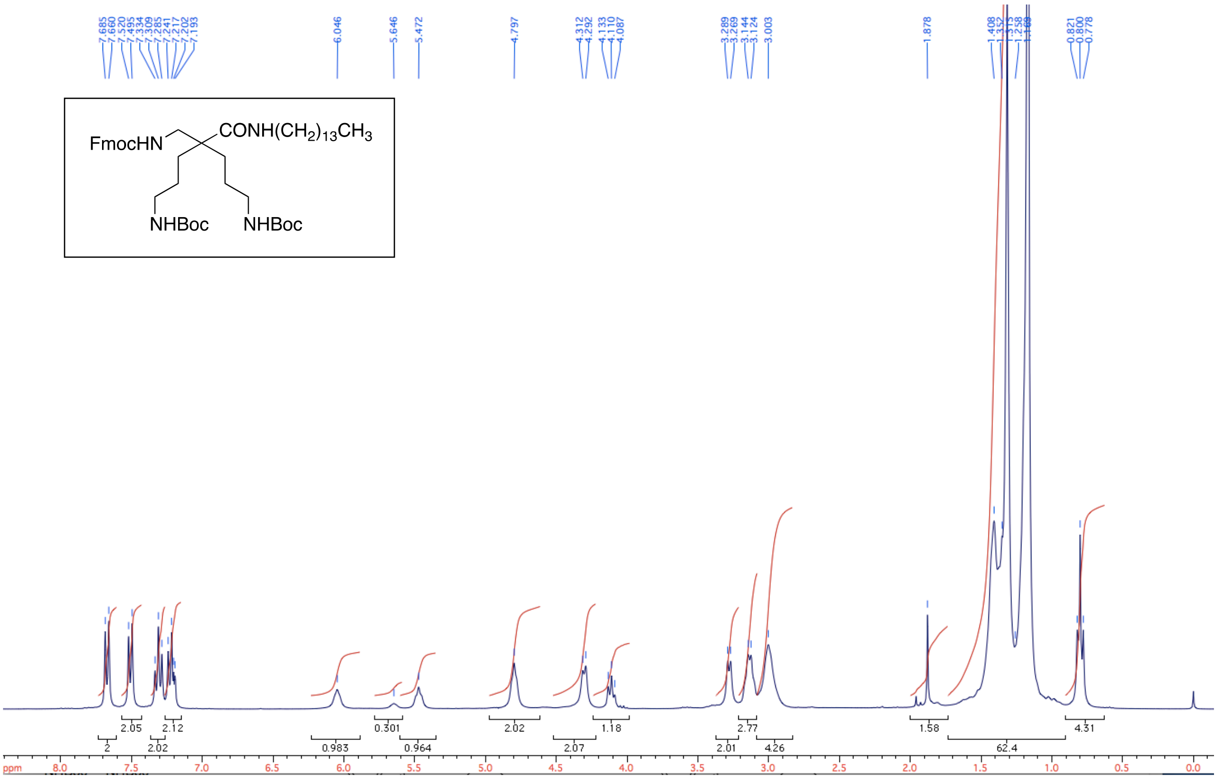

^{13}C NMR spectra of Fmoc-β^{2,2} *h* bis-Orn(Boc)$_2$NH(CH$_2$)$_{13}$CH$_3$, 300 MHz, CDCl$_3$

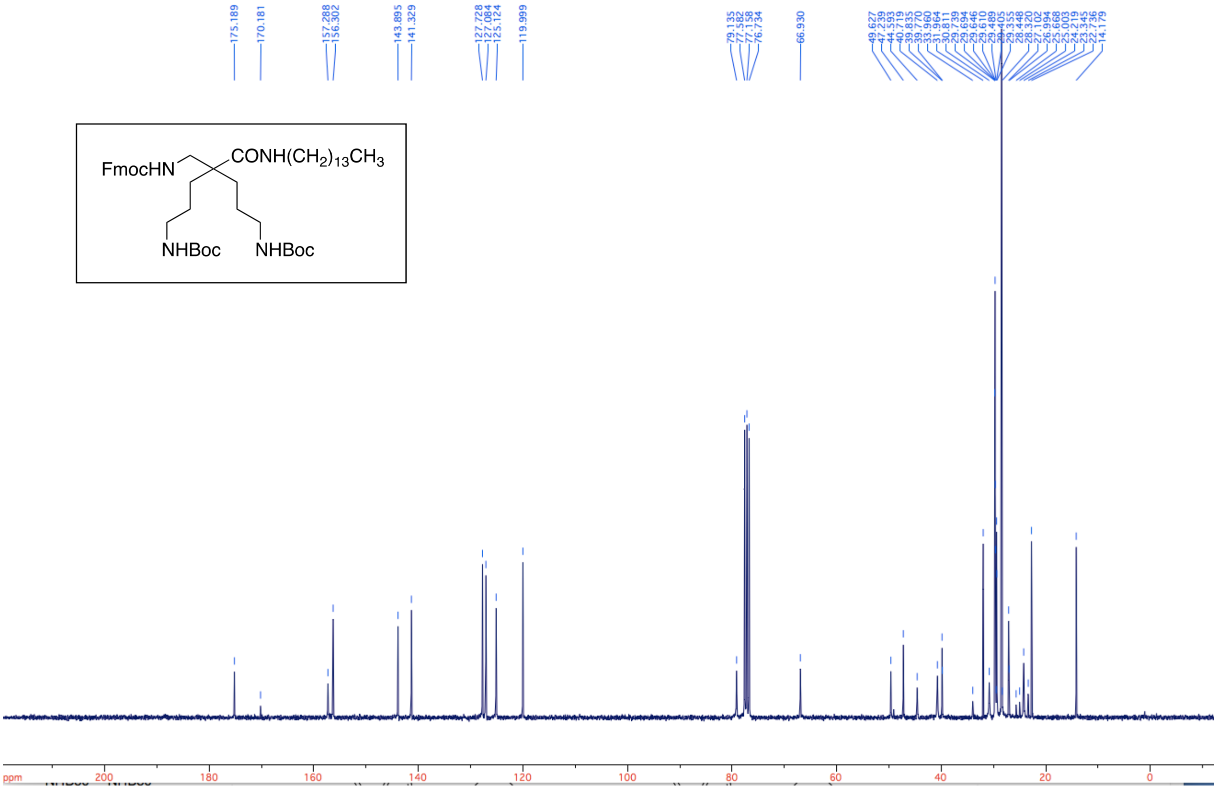

^{1}H NMR spectra of H-β[2,2] *h* bis-Orn(Boc)[2]NH(CH[2])^{13}CH[3] 31, 300 MHz, CD[3]OD

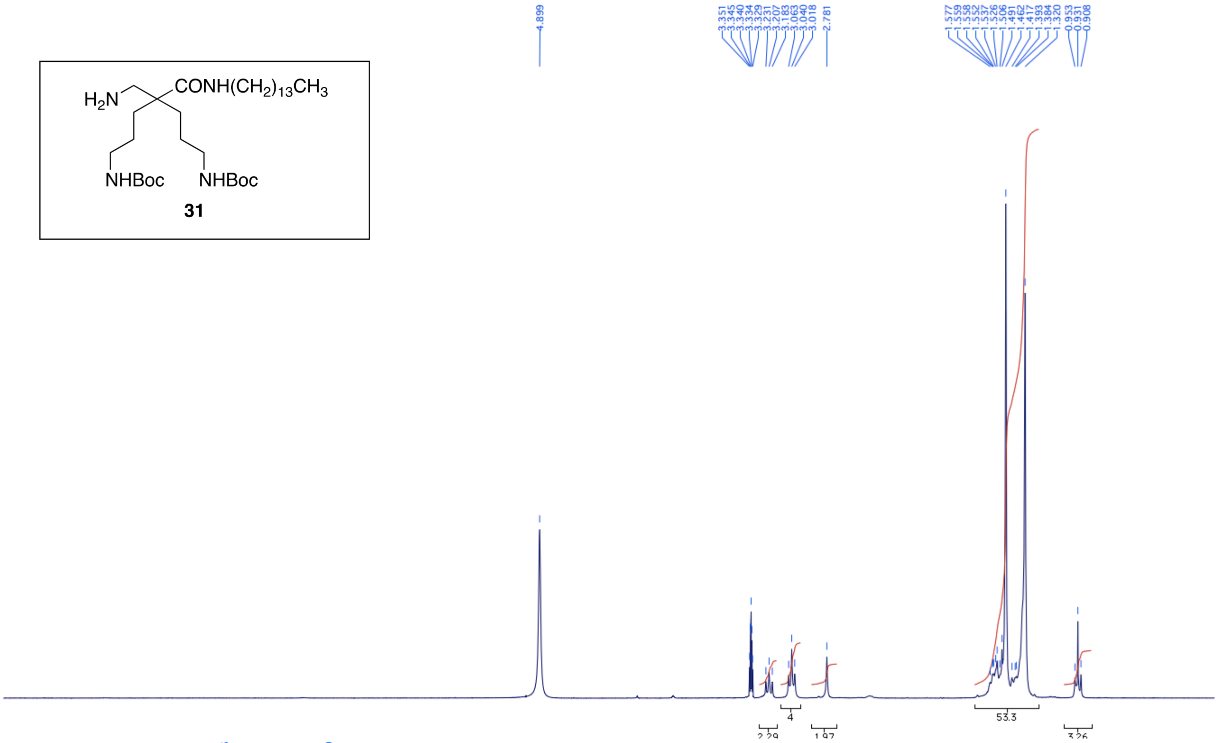

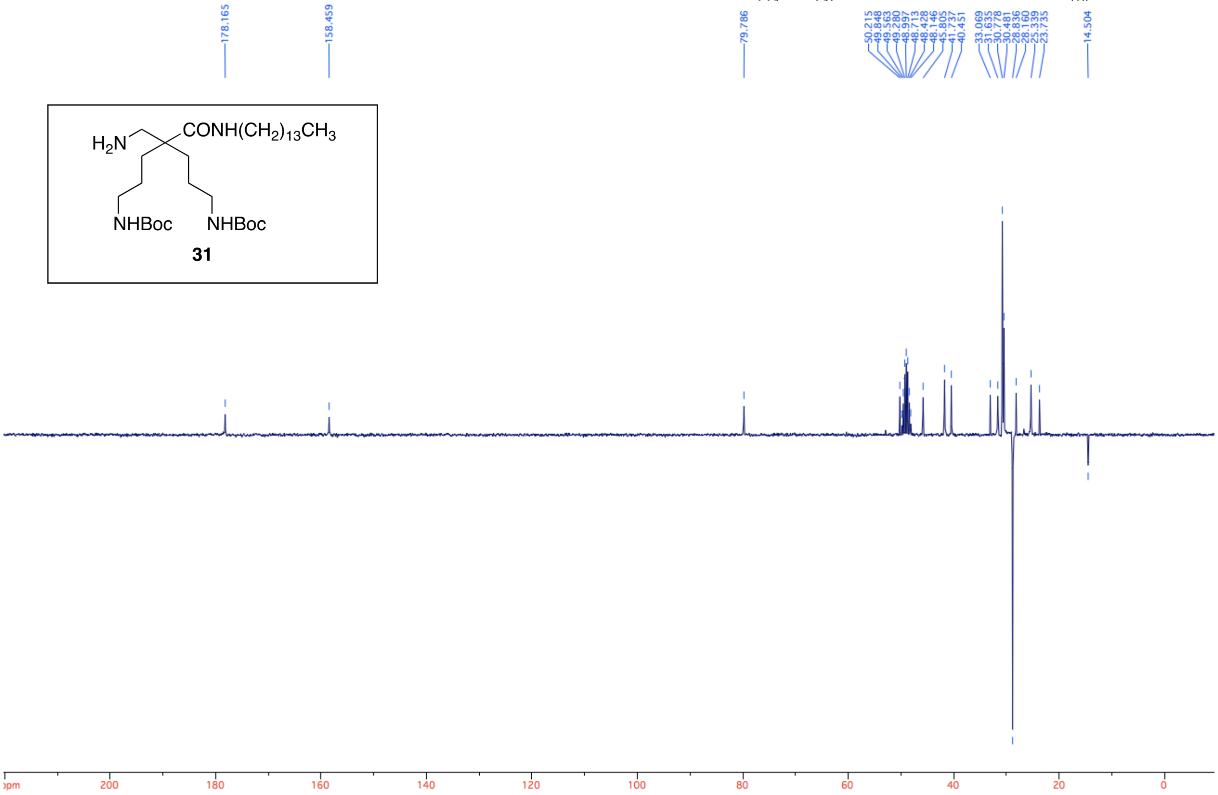

^{1}H NMR spectra of Fmoc-Tbt-$\beta^{2,2}$ *h* bis-Orn(Boc)$_2$-NH(CH$_2$)$_{13}$CH$_3$ 32, 300MHz, CD$_3$OD

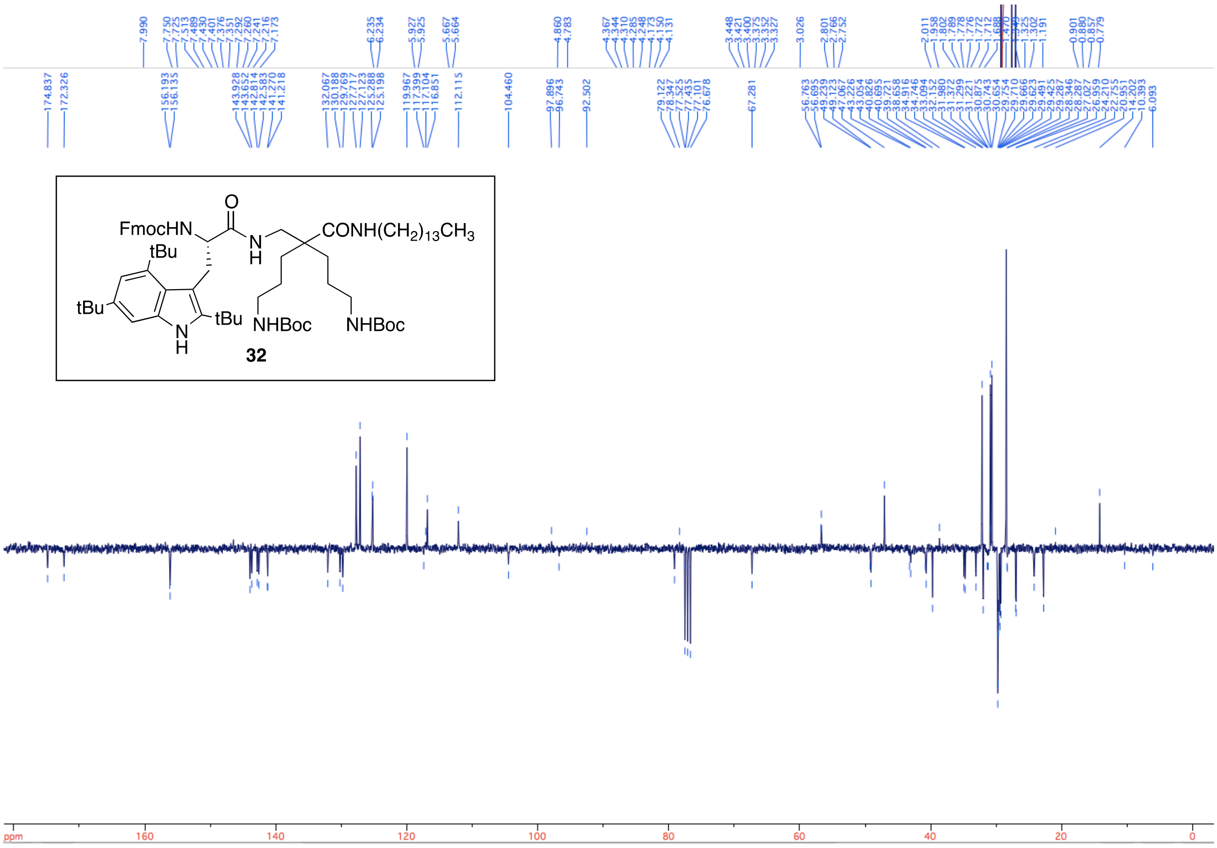

Supplement: Supplementary file 1 [file molecules-24-01702-s001.pdf]
